# pH-Dependent Antioxidant Mechanisms of Harmalol Toward HOO^•^ Radicals in Aqueous Solution: A Quantum Chemical Study

**DOI:** 10.3390/ijms27135959

**Published:** 2026-07-02

**Authors:** Agnieszka Kowalska-Baron

**Affiliations:** Institute of Natural Products and Cosmetics, Faculty of Biotechnology and Food Sciences, Lodz University of Technology, Stefanowskiego 2/22, 90-537 Lodz, Poland; agnieszka.kowalska-baron@p.lodz.pl

**Keywords:** harmalol, ß-carbolines, FHT, SET, RAF, PCET, antioxidant mechanisms, pKa

## Abstract

Harmalol is a β-carboline alkaloid exhibiting promising antioxidant properties; however, a comprehensive understanding of its radical scavenging mechanisms in aqueous media across a wide pH range remains limited. In this study, the antioxidant activity of harmalol toward hydroperoxyl radicals was investigated theoretically at the M06-2X/6-311+G(d,p)/PCM(water) level by combining thermodynamic and kinetic analyses over the pH range 2–13. The calculations revealed that the antioxidant behavior of harmalol strongly depends on its protonation state, tautomeric form, and the surrounding pH. Under physiological conditions, the monocationic form predominates, with a smaller contribution from the neutral/zwitterionic I and II species, and radical scavenging proceeds predominantly via proton-coupled electron transfer (PCET)-type hydrogen-transfer reactions involving the monocationic, neutral and zwitterionic I forms as well as radical adduct formation (RAF) mechanism involving zwitterion I. Analysis of SOMO distributions, spin densities, and atomic charges confirmed that the hydrogen transfer reactions for monocationic, neutral and zwitterionic I forms do not follow a classical hydrogen atom transfer (HAT) mechanism. The zwitterion I and neutral forms of harmalol exhibited significantly higher apparent rate constants for the PCET reaction than the monocationic species. Under alkaline conditions, the monoanionic forms exhibit the most favorable thermodynamic parameters toward radical scavenging via formal hydrogen transfer mechanism. Relaxed potential energy surface scans suggest that hydrogen transfer from both monoanionic forms may proceed through a barrierless pathway, while radical adduct formation can also contribute to the antioxidant activity under strongly basic conditions. In addition, monoanion II efficiently participates in single-electron transfer (SET) reactions characterized by very high apparent rate constants. Overall, the results demonstrate that the antioxidant efficiency of harmalol increases with increasing pH and provide detailed insight into the pH-dependent radical scavenging mechanisms of β-carboline derivatives in aqueous environments.

## 1. Introduction

Reactive oxygen species (ROS), which belong to the group of free radicals, are characterized by the presence of an unpaired electron in the valence shell or in the excited state. As a result, they are highly reactive and tend to achieve stability by accepting a hydrogen atom or an electron. ROS are naturally produced in the human body during fundamental metabolic processes (endogenous sources) and play crucial roles in immune defense and intracellular signaling [1,2]. At low to moderate levels, ROS are essential for maintaining normal physiological functions [3,4,5].

However, ROS may also originate from external sources, including environmental pollution, radiation, and xenobiotics [4,5,6,7]. Human body has various defense systems to neutralize these reactive species, but these mechanisms are not always sufficient. Consequently, excessive accumulation of ROS in living organisms can lead to oxidative stress. Under such conditions, ROS trigger a cascade of damaging reactions that affect vital biological components such as DNA, proteins, and lipids, contributing to the development of numerous diseases, including cancer, diabetes, atherosclerosis, and neurodegenerative disorders [4,5,6]. To safeguard critical biomolecules from free radical-induced damage and to preserve the balance between ROS production and elimination (neutralization), supplementation with natural antioxidants is often recommended [4,6].

Antioxidants are compounds capable of inhibiting the production of ROS and/or scavenging them, thereby limiting oxidative damage. They can be broadly classified into three groups based on their mechanism of action: type I antioxidants, which directly react with free radicals; type II antioxidants, which prevent ROS formation (e.g., via metal chelation, UV absorption, or inhibition of ROS-generating enzymes) and type III, which are involved in the repair of oxidatively damaged biomolecules [8,9]. Among these, type I antioxidants, known as chain-breaking antioxidants, play a central role in direct radical neutralization through various mechanisms such as formal hydrogen transfer (FHT) or single electron transfer (SET). These processes may occur either directly (FHT, SET) or through stepwise pathways via sequential proton loss (SPL-FHT, SPL-ET) or via SET-PT (proton transfer) mechanisms, ultimately leading to the formation of more stable and less harmful radical products. Additionally, less reactive species can arise through direct interactions between radicals and antioxidants via the radical adduct formation (RAF) mechanism [8,9].

A majority of primary antioxidants are weakly acidic, therefore multiple acid-base species can coexist in water at a given pH. The abundance of these species is governed by their chemical structure as well as the pH of the medium. Changes in pH affect the protonation state of antioxidant molecules, thereby altering their electronic structure, reactivity, and preferred free radical scavenging mechanisms. For example, deprotonated species are expected to exhibit electron-related channels, whereas neutral species may preferentially undergo processes involving hydrogen or proton transfer pathways [8,9].

Reactions involved in primary antioxidant modes of action should be spontaneous and fast enough. Spontaneity of the reaction depends on thermodynamic feasibility given by Gibbs free energy (ΔG). Highly endergonic reactions are generally thermodynamically disfavored and are therefore unlikely to contribute significantly to antioxidant activity. Exergonic reactions can proceed at either fast or slow rates. On the other hand, moderately endergonic reactions (ΔG < 10 kcal/mol), may still contribute significantly to antioxidant capacity, particularly when the reaction barrier is low. Additionally, for some SET reactions thermochemical and kinetic data may exhibit opposing trends: endergonic SET reactions may proceed with relevant rate, and on the other hand: highly exergonic SET reactions can fall within the inverted region of Marcus parabola, where the reaction barriers increase as the Gibbs energies become more negative. As a result, even strongly exergonic processes may proceed slowly, whereas moderately endergonic reactions can occur at appreciable rates [5,8,9]. Consequently, a comprehensive evaluation of antioxidant activity requires consideration of both thermodynamic descriptors and kinetic parameters [9].

Several experimental protocols have been developed to evaluate antioxidant capacity, including DPPH^•^ (2,2-diphenyl-1-picrylhydrazyl) assays, ABTS^•+^ (2,2′-azinobis(3-ethylbenzthiazoline-6-sulfonic acid) assay, ORAC (oxygen radical absorbance capacity) assay, TRAP (total radical-trapping antioxidant parameter) assay, FRAP (ferric reducing antioxidant power) assay, and the Folin–Ciocalteu reagent assay. However, none of these approaches provides a universal, fully quantitative, and directly comparable measure of antioxidant activity. This limitation arises from fundamental differences in assay conditions, such as solvent type, pH and reaction time [8,10].

Moreover, each assay may be governed by distinct dominant antioxidant mechanism. For instance, formal hydrogen atom transfer (FHT) is the primary free radical scavenging pathway in ORAC and TRAP assays, whereas the FRAP is based on single electron transfer (SET) [8]. DPPH^•^ and ABTS^•+^ assays are primary based on SET but may also involve FHT [10]. As a result, the measured antioxidant activity of a given compound may vary significantly depending on the assay employed. In some cases, one method may provide a reliable assessment, while another may lead to misleading conclusions regarding relative antioxidant potency.

Furthermore, the reactivity of an antioxidant toward free radicals is strongly influenced by environmental factors, including solvent polarity and pH, which affect both the speciation and concentration of the active forms. Consequently, differences in experimental conditions can alter not only the dominant reaction mechanism but also the observed activity trends across a series of compounds. The lack of standardized reporting units, such as the frequent use of Trolox equivalents or IC_50_ values, further complicates direct comparisons between studies and theoretical predictions [11].

Regarding the assessment of antioxidant activity in vitro using cell culture models, this methodology has several limitations. One of these is related to the compartmentation of compounds within the cellular system, particularly in the case of lipophilic molecules. It is often implicitly assumed that the concentration of a tested compound is uniform across all compartments and equal to its initial concentration in the culture medium; however, this assumption is incorrect, as compounds may distribute unevenly between intracellular and extracellular compartments, thereby potentially leading to misinterpretation of the antioxidant capacity of the tested compound [6]. For the above-mentioned reasons, the selection of an appropriate experimental assay should be guided by prior understanding of the underlying reaction mechanisms relevant to the compound under investigation.

In this context, theoretical approaches using methods of computational quantum chemistry provide valuable complementary insight into antioxidant mechanism. Advances in computational chemistry have made it possible to reliably evaluate both thermodynamic and kinetic aspects of antioxidant activity [5,8,9,11]. In particular, theoretical methods enable consideration of several important effects such as quantum tunneling as well as pH-dependent speciation, which are often difficult to capture experimentally [5,8,9]. The most frequently applied theoretical approach to study antioxidant potency of medium-size molecules is Density Functional Theory (DFT) with B3LYP, M05-2X and M06-2X exchange-correlation functional [9]. Among these the M06-2X functional has been shown to provide reliable description of thermodynamics and kinetics of the reactions involved in the antioxidant mechanisms [12].

In this study, the M06-2X/6-311+G(d,p)/PCM(water) method was applied to investigate the antioxidant mechanisms of harmalol (1-methyl-4,9-dihydro-3H-pyrido [3,4-b]indol-7-ol), a naturally occurring β-carboline alkaloid found in plants such as *Peganum harmala*. β-Carboline alkaloids have attracted considerable attention owing to their broad spectrum of biological activities, including antibacterial, antiviral, analgesic, antioxidant, anticancer, and neuroprotective effects [13,14,15,16,17,18,19]. In particular, harmalol has been reported to stimulate cellular melanogenesis and enhance tyrosinase activity, suggesting its potential therapeutic application in the treatment of hypopigmentation disorders such as vitiligo [20]. Furthermore, harmalol exhibits moderate yet selective affinity toward several central nervous system receptors, indicating its potential neuromodulatory activity [21].

In addition, harmalol and related dihydro-β-carbolines have been suggested to protect against oxidative neuronal damage, although the molecular basis of their antioxidant activity remains insufficiently understood [19].

Harmalol may be classified as both a primary (chain-breaking) and secondary (preventive) antioxidant. Its antioxidant activity has been experimentally demonstrated [16]. Among five β-carboline alkaloids—harmine, harmaline, harmalol, harmane, and 1,2,3,4-tetrahydroharmane-3-carboxylic acid—harmalol exhibited the highest antioxidant activity in ABTS^•+^, FRAP, and reducing power assays [16].

Moreover, harmalol, similarly to other harmala alkaloids, inhibits monoamine oxidase (MAO). Inhibition of this enzyme suppresses excessive production of H_2_O_2_, thereby contributing to the attenuation of oxidative stress [17,18].

To date, only limited studies are available addressing the antioxidant mechanisms of harmalol. In a previous study [16], global reactivity descriptors (electronegativity, chemical potential, hardness, softness, and electrophilicity index) were calculated at the B3LYP/6-31+G(d,p) level of theory. Based on intrinsic thermochemical parameters such as bond dissociation enthalpy (BDE(N–H)), ionization potential (IP), proton dissociation enthalpy (PDE), proton affinity (PA), and electron transfer enthalpy (ETE)), which are commonly used as descriptors of the FHT, SET–PT, and SPL-ET mechanisms, respectively, it was proposed that harmalol scavenges free radicals predominantly via the SET–PT mechanism. However, it should be noted that the theoretical model did not account for solvent effects, and only the radical formed via hydrogen abstraction from the pyrrolic N–H group of harmalol was considered.

More recently, comprehensive experimental and theoretical studies have focused on the acid–base speciation, tautomeric equilibria, and photophysical properties of harmalol in aqueous solution [22,23]. In the work of Villaruel et al. [22], the UV–Vis absorption spectra of the different acid–base and tautomeric species were investigated using TD-DFT calculations at the B3LYP/aug-cc-pVDZ level of theory, providing a detailed spectroscopic characterization and assignment of the individual forms of harmalol over a wide pH range.

To date, a comprehensive understanding of the antioxidant mechanism of harmalol in aqueous environments remains lacking, particularly with respect to studies integrating both thermodynamic and kinetic perspectives across a wide pH range. Moreover, the role of different protonation states of harmalol in modulating its reactivity toward HOO^•^ radicals has not been systematically explored.

HOO^•^ was selected in this study as the model reactive oxygen species for several reasons. HOO^•^ is the smallest member of the peroxyl radical family and is considered an important reactive oxygen species involved in oxidative processes associated with aerobic metabolism [24]. In contrast to the hydroxyl radical (^•^OH), which reacts with most biomolecules at nearly diffusion-controlled rates and is therefore difficult to scavenge, HOO^•^ has a sufficiently long lifetime to be intercepted by antioxidants before causing oxidative damage to biologically relevant targets [9,11].

Moreover, from a theoretical perspective, HOO^•^ is particularly suitable for kinetic studies because transition states involving HOO^•^ generally exhibit a lower multireference character than those involving more reactive radicals, allowing reliable treatment with conventional DFT methods [11]. Consequently, HOO^•^ has been widely employed as a model radical in computational studies of antioxidant activity and radical-scavenging mechanisms.

Although O_2_^−•^ is the predominant species under physiological and alkaline conditions, it is generally less reactive toward formal hydrogen atom transfer (FHT) processes than its protonated counterpart, HOO^•^ [9]. Furthermore, as discussed by Galano [11], when comparing theoretical rate constants obtained for HOO^•^ with experimental data at a given pH, the calculated values should be corrected by the molar fraction of HOO^•^ present under those conditions. For example, at pH 7.4 the apparent rate constant should be weighted by the corresponding fraction of HOO^•^ in the HOO^•^/O_2_^−•^ equilibrium [11].

This study aims to provide a detailed theoretical investigation of the antioxidant activity of harmalol towards HOO^•^ radicals at the M06-2X/6-311+G(d,p)/PCM(water) level of theory, taking into account various protonation states and tautomeric forms of this compound occurring across the pH range of 2–13. Based on a combined analysis of thermodynamic and kinetic parameters (Gibbs free energies and reaction rate constants), the most probable mechanism of free radical scavenging by harmalol under these conditions is identified. The obtained results are expected to provide deeper insight into the structure–activity relationships governing the antioxidant potential of structurally related compounds.

## 2. Results and Discussion

### 2.1. Acid–Base Speciation, pKa Prediction, and Relative Stability of Harmalol

Depending on the pH of the medium, harmalol may exist in several acid–base and tautomeric forms in aqueous solution. Previous experimental and theoretical studies have identified monocationic, neutral, zwitterionic, and monoanionic species, whose relative abundances depend strongly on pH [22,23,25,26]. In particular, more than one zwitterionic and monoanionic form has been reported, differing in the localization of the negative charge either on the pyrrolic nitrogen or within the phenoxide moiety [22]. Under extremely acidic (pH < 2) and highly alkaline conditions (pH > 16), dicationic and dianionic species have also been proposed [26,27,28]; however, these species are not expected to contribute significantly under the conditions considered in the present study and were therefore not investigated further.

**Figure 1:**
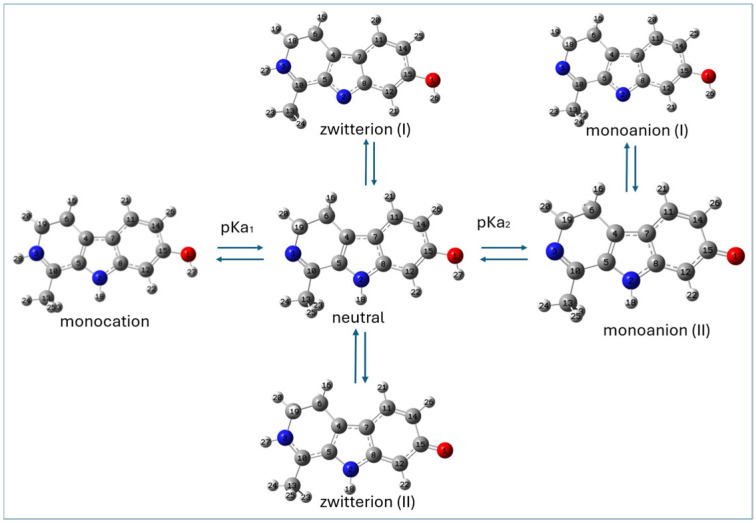
M06-2X/6-311+G(d,p)/PCM(water) optimized structures of possible forms of harmalol in the ground state at the 2–13 pH range. Atomic labels according to Gaussian 09.

The M06-2X/6-311+G(d,p)/PCM(water) optimized structures of the harmalol species considered in this work are presented in Figure 1.

The existence of these forms is supported by UV–Vis spectroscopic measurements combined with quantum-chemical calculations [22]. Detailed spectroscopic characterization and assignment of the absorption bands have been reported elsewhere and will not be repeated here. Notably, the absorption spectrum of the monocationic species extends throughout the UVB and UVA regions, suggesting a potential photoprotective role through the absorption of UV radiation. In addition, previous photophysical studies [22,23] demonstrated that harmalol exhibits negligible or extremely low photosensitized ROS generation, including singlet oxygen and hydrogen peroxide formation upon photoexcitation. These findings suggest that harmalol may absorb UV radiation without promoting significant photoinduced oxidative stress.

The distribution of harmalol species in aqueous solution is governed by the corresponding acid–base equilibria and their associated pKa values. Alomar et al. [26] reported two acid–base equilibria in the pH range of 2–13. The first equilibrium, between the monocationic and neutral species, is associated with deprotonation of the pyridinic nitrogen and is characterized by a pKa_1_ value of 8.5. The second equilibrium corresponds to deprotonation of the hydroxyl group (pKa_2_ = 11.2), leading to the formation of the monoanionic II species. Similar values (pKa_1_ = 8.5 and pKa_2_ = 11.3) were subsequently reported by Villarruel et al. [22]. The theoretically predicted pKa values of harmalol, obtained using the parameter-fitting methodology developed by Galano et al. [29] at the M06-2X/6-311+G(d,p)/PCM level (see Table 1), are in excellent agreement with the experimentally determined values reported in the literature, with deviations lower than 0.5 pKa units. The calculated pKa_1_ value of 8.4 is nearly identical to those reported by Villarruel et al. [22] and Alomar et al. (8.5) [26]. Similarly, the calculated pKa_2_ value of 10.8 shows good agreement with the literature values of 11.2 [26], 11.3 [22] and 11.5 [25]. These results demonstrate the reliability of the parameter-fitting approach proposed by Galano et al. [29] for the β-carboline under study.

The studies reported by Villarruel et al. [22] demonstrated that the acid–base behavior of harmalol is more complex than previously assumed, involving tautomeric equilibria between the neutral and zwitterionic species, zwitterion I and zwitterion II, as well as between two monoanionic forms, monoanion I and monoanion II, differing in the localization of the negative charge either on the pyrrolic nitrogen or within the phenoxide moiety, respectively. Consequently, several species may coexist in solution depending on pH, and their relative populations are determined by both acid–base and tautomeric equilibria. The relative Gibbs free energies of harmalol species involved in the neutral–zwitterionic and monoanionic tautomeric equilibria are summarized in Table 2. The neutral form was found to be thermodynamically preferred over both zwitterionic species, whereas monoanion II exhibited the lowest Gibbs free energy among the deprotonated forms. These results are consistent with previous theoretical studies [22].

Experimentally determined pKa values were used to estimate the molar fractions of harmalol species at different pH values. At physiological pH (7.4), the monocationic form predominates (92.64%), whereas the neutral/zwitterionic species account for 7.36% of the total population and the monoanionic forms are negligible (0.001%).

At pH 2, the monocationic form is the only species present, whereas at pH 13, the monoanionic forms predominate (98.44%), with a minor contribution from the neutral/zwitterionic species (1.56%) and negligible amounts of the monocation.

At pH < pKa_1_, the monocationic species predominate over the neutral/zwitterionic species, whereas at pH > pKa_1_, the neutral/zwitterionic species become predominant. Similarly, at pH > pKa_2_, the monoanionic forms predominate over the neutral/zwitterionic species. It should be noted that the reported molar fractions correspond to the overall protonation states rather than individual tautomers, since the available experimental pKa values describe the overall acid–base equilibria of harmalol.

### 2.2. Preliminary Assessment of Antioxidant Activity of Harmalol Based on Global Reactivity Descriptors and Intrinsic Thermochemical Parameters

The determined global reactivity descriptors, including ionization potential (IP), electron affinity (EA), electronegativity (χ), chemical potential (μ), global hardness (η), global softness (S), and electrophilicity (ω) together with electron-donating power (ω−) and electron-accepting power (ω+) are summarized in Table 3. These descriptors provide insight into the intrinsic electron-donating and electron-accepting tendencies of harmalol, which are closely related to its antioxidant potential, particularly in electron transfer–based mechanisms.

As shown in Table 3, deprotonation of the monocationic form of harmalol leads to a decrease in ionization potential (IP), electrophilicity (ω), electronegativity (χ), as well as electron-donating (ω−) and electron-accepting (ω+) powers. Lower values of IP, χ and ω indicate an increased tendency of the molecule to act as an electron donor in interactions with radical species, while the reduction in electron-accepting power (ω+) and electron affinity (EA) suggests a diminished tendency to accept electrons.

The calculated chemical potentials further support this trend. The monoanionic species exhibit the highest (least negative) μ values among the investigated forms, indicating an increased tendency to donate electron and participate in electron-transfer processes.

The zwitterionic species exhibit distinct reactivity patterns compared with the neutral form. Although they possess higher electronegativity and electrophilicity indices, both zwitterions are characterized by lower ionization potential and lower hardness values than the neutral species. The reduced IP values indicate that electron transfer from the zwitterionic species is thermodynamically more favorable, while the lower hardness suggests an increased susceptibility to changes in electron density. Among the two zwitterionic forms, zwitterion II exhibits lower ionization potential and hardness, indicating a slightly greater propensity for electron-transfer processes.

Overall, successive deprotonation enhances the electron-donating properties of harmalol. The monoanionic forms are expected to be the most effective electron donors and thus the most reactive species in the SET mechanism, whereas this pathway is likely of minor importance for the monocationic form.

The M06-2X/6-311+G(d,p)/PCM(water) calculated values of adiabatic ionization potential (AIP), bond dissociation enthalpy (BDE), proton dissociation enthalpy (PDE), proton affinity (PA), and electron transfer enthalpy (ETE), which are commonly used as primary descriptors of the SET, FHT, SET–PT, and SPL-ET mechanisms, are summarized in Table 4.

As shown in Table 4, relatively low BDE values (compared to AIP values) associated with radicals arising from homolytic cleavage of the phenolic O–H bond indicate that the formal hydrogen transfer (FHT) mechanism from the hydroxyl group is a probable mode of antioxidant activity. The BDE values corresponding to radicals formed via H abstraction from the O–H group are lower than those associated with N–H bond cleavage, suggesting that the former radicals are more stable.

**Figure 2:**
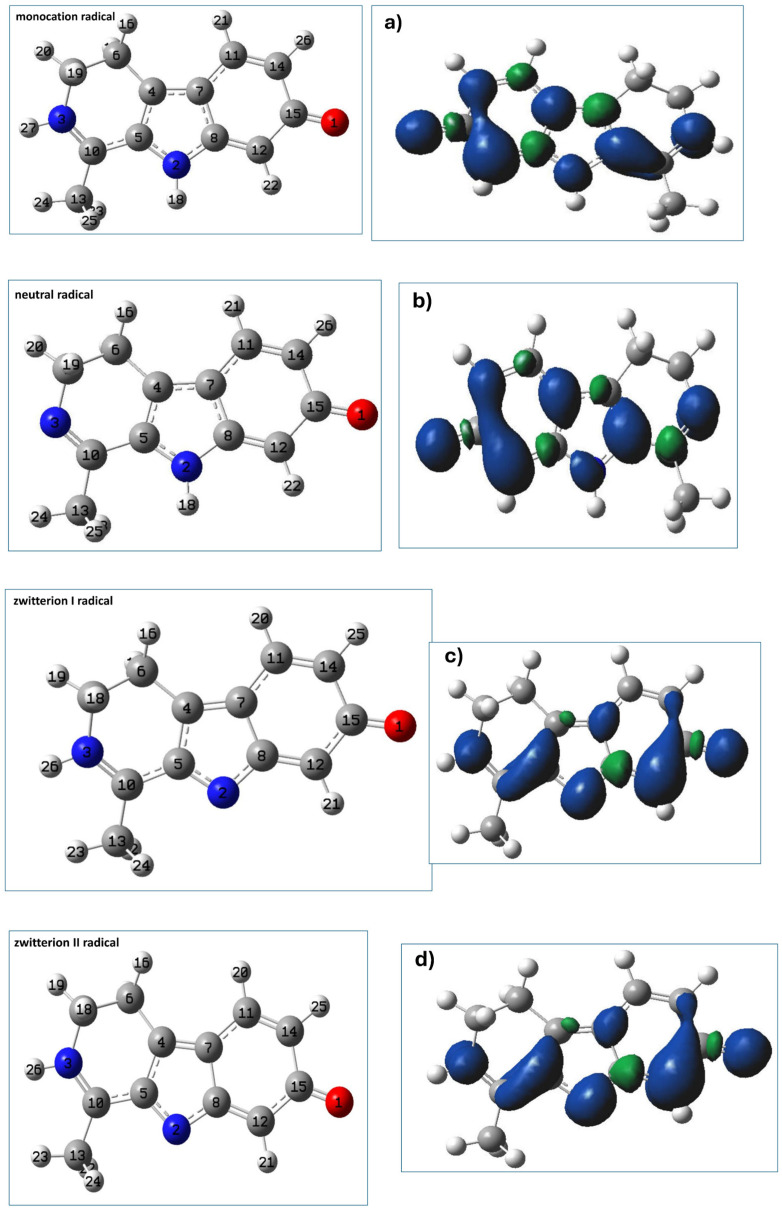

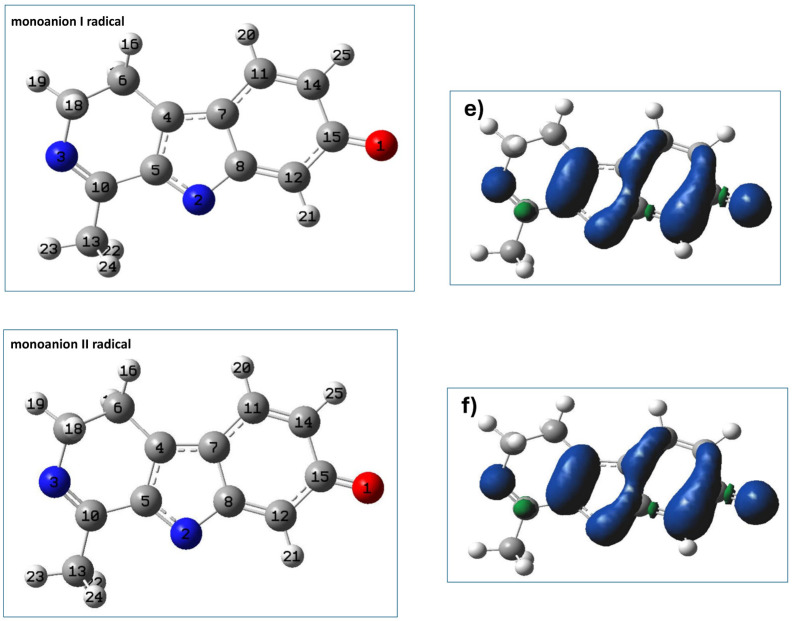
M06-2X/6-311+G(d,p)/PCM(water) calculated geometries (left panel) of the most stable antioxidant radicals of: (**a**) monocationic, (**b**) neutral, (**c**,**d**) zwitterionic I and II, respectively and (**e**,**f**) monoanionic I and II, respectively, species of harmalol, together with their spin density distributions (right panel, isovalue 0.002). Atomic labels according to Gaussian 09.

Graphical representations of the most stable radical species of harmalol, formed via H abstraction from the O–H group (monocation, neutral, zwitterion I, monoanion I) and from the N–H group (zwitterion II and monoanion II), calculated at the M06-2X/6-311+G(d,p)/PCM(water) level, are shown in Figure 2, together with their spin density distributions. The spin density distributions (Figure 2, right panel) show significant delocalization over the entire aromatic system, which contributes to the enhanced stability of the formed radicals, supporting the feasibility of the FHT mechanism.

The proton dissociation tendency of harmalol is characterized by the calculated proton affinity (PA). The PA values obtained for the monocationic and neutral forms of harmalol are lower than the corresponding BDE values (Table 4), suggesting that these species are more prone to undergoing the SPL mechanism, which may be followed by ET (SPL-ET) or FHT (SPL-FHT) pathways.

The adiabatic ionization potential (AIP) serves as a fundamental descriptor of the SET mechanism, representing the minimum energy required to remove an electron from the antioxidant and form a radical cation. Lower AIP values reflect an increased propensity for electron donation and thus enhanced antioxidant activity via the SET pathway.

The relatively high AIP values of the monocationic and neutral forms of harmalol indicate that the SET step is thermodynamically unfavorable, suggesting that electron transfer is not a competitive pathway for these species. Although the SET–PT mechanism cannot be completely excluded for the neutral form (PDE = 11.11 kcal/mol, Table 4), the high adiabatic ionization potential suggests that this pathway is unlikely to be operative.

In contrast, the lowest AIP values observed for the monoanionic form II indicates that this species is a good electron donor. Moreover, moderate ETE values suggest an increased propensity for electron transfer, making the SET mechanism potentially competitive for this form.

A commonly adopted criterion for SET feasibility is that the electron affinity (EA) of the reacting radical exceeds the ionization potential of the antioxidant [5,9]. The EA of a radical (R^•^) is defined as the enthalpy change associated with the reaction R^•^ + e^−^ → R^−^ [30]. The M06-2X/6-311+G(d,p)/PCM(water) calculated EA value for the HOO^•^ radical is −71.858 kcal/mol [31], indicating that the energy released by HOO^•^ radical upon electron attachment is insufficient to compensate for the AIP of the monoanionic form.

It should be noted, however, that thermochemical calculations of EA require the enthalpy of a free electron, which cannot be directly determined and must be approximated using reference values, introducing some uncertainty [9]. Previously reported data suggest that the energy released upon electron attachment to other radicals (e.g., NO_2_^•^, HO^•^, SO_4_^•−^, CH_3_O^•^, and CCl_3_O^•^) may be sufficient to compensate for the AIP of the monoanionic II form of harmalol [31].

Notably, intrinsic thermochemical parameters do not account for the specific characteristics of the reacting radical. Therefore, to obtain a more realistic description of antioxidant activity, it is necessary to evaluate reaction Gibbs free energies (ΔG) for interactions with relevant radical species, such as HOO^•^. Furthermore, these descriptors do not provide direct insight into radical adduct formation (RAF) pathways, which involve the formation of new covalent bonds. Consequently, the assessment of RAF mechanisms requires explicit calculation of reaction Gibbs free energies for adduct formation. These observations highlight the importance of considering explicit reaction thermodynamics when evaluating antioxidant mechanisms.

### 2.3. Thermodynamic Analysis of Harmalol Reactions with HOO^•^ Radical

The Gibbs free energies associated with the reactions involving harmalol and HOO^•^ radicals related to FHT, RAF, SET, SET-PT, SPL-ET, and SPL-FHT mechanisms are summarized in Table 5. The corresponding relative electronic energies (ΔE), enthalpies (ΔH), and entropies (ΔS) are reported in the Supplementary Material (Tables S1–S3).

The most stable radical adducts formed via the RAF mechanism correspond to the addition of the HOO^•^ radical at the C_4_ position of harmalol (see Supplementary Material, Table S4).

The calculated Gibbs free energy changes (ΔG) indicate that the RAF reactions of the monocationic and monoanionic species are weakly exergonic, whereas for neutral and zwitterionic II are slightly endergonic. Notably, the addition of the HOO^•^ radical to zwitterion I is markedly exergonic, indicating a particularly favorable radical adduct formation pathway for this species.

The results of the M06-2X/6-311+G(d,p)/PCM(water) calculations (Table 5) showed that, within the FHT mechanism, hydrogen abstraction from the OH group is more favorable than from the N–H bond of the pyrrolic moiety. As shown in Table 5, formal hydrogen transfer (FHT) reactions are exergonic for all considered forms of harmalol, and each successive deprotonation facilitates the FHT process, as reflected by decreasing ΔG values. It should be noted that for the neutral and monoanionic forms, the Gibbs free energy of the FHT reaction is identical to that of the second step of the SPL–FHT mechanism.

The single electron transfer (SET) mechanism appears to be feasible only for the monoanionic forms of harmalol, as the corresponding ΔG value is moderately endergonic (<10 kcal/mol). In contrast, for the neutral, zwitterionic and monocationic forms, SET is endergonic (>10 kcal/mol) and thus thermodynamically unfavorable.

As shown in Table 5, proton dissociation steps (SPL) are exergonic. Deprotonation from the pyridinic N–H group in the monocation is more exergonic than the corresponding proton loss from the O–H group in the neutral form.

The SET–PT mechanism is unlikely to be operative for harmalol, as the first step for both neutral and monocationic forms is highly endergonic (>10 kcal/mol). This finding contrasts with previous work by Senhaji et al. [16], in which the SET–PT mechanism was proposed as the dominant pathway for free radical scavenging. However, in that study, the preferred antioxidant mechanism was inferred solely from intrinsic thermochemical descriptors (BDE, IP, PDE, PA, and ETE), without accounting for solvent effects and pH conditions. Additionally, only radicals formed via hydrogen abstraction from the pyrrolic N–H group were considered. In contrast, the present M06-2X/6-311+G(d,p)/PCM(water) calculations indicate that hydrogen transfer from the pyrrolic N–H group in the monocationic and neutral forms is endergonic (ΔG = +5.21 and +1.11 kcal mol^−1^, respectively). Therefore, the phenolic O–H group constitutes the preferred reactive site for FHT in these species, and only this pathway was considered in the kinetic analysis.

Kinetic investigations were primarily performed for the thermodynamically preferred forms within each protonation state, namely the monocationic, neutral, and monoanionic II species, since the overall antioxidant activity depends not only on the intrinsic reactivity of a given species but also on its relative abundance in solution.

In addition, selected alternative tautomers exhibiting particularly favorable thermodynamic characteristics toward radical scavenging were also subjected to kinetic analysis. Specifically, zwitterion I was investigated because it exhibited the most favorable thermodynamic parameters among the zwitterionic species, with both RAF and FHT pathways being strongly exergonic. Monoanion I was additionally considered owing to its particularly favorable thermodynamic characteristics toward formal hydrogen transfer FHT. In contrast, zwitterion II was included in the thermodynamic analysis but was not further investigated kinetically, as its calculated reactivity was lower than that of zwitterion I.

Accordingly, the RAF and FHT pathways of the monocationic, neutral, zwitterionic I, monoanionic I, and monoanionic II forms of harmalol, as well as the SET mechanism of the monoanionic species, were further analyzed within a kinetic framework.

### 2.4. Kinetics of Reactions Involved in RAF, FHT and SET Mechanisms

**Figure 3:**
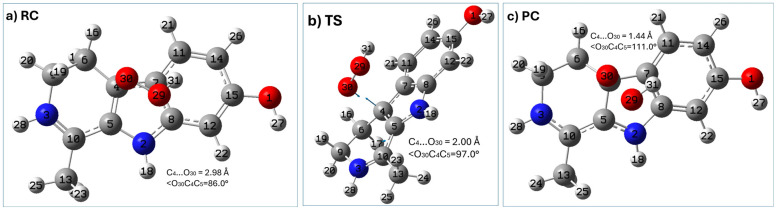
Graphical representations of the stationary points: (**a**) reactant complex (RC), (**b**) transition state (TS) and (**c**) product complex (PC) encountered along the RAF reaction pathway of harmalol (monocation) with hydroperoxyl radicals. Displacement vectors of imaginary frequencies at 610.58*i* cm^−1^ are shown as blue arrows. Atomic labels according to Gaussian 09.

**Figure 4:**
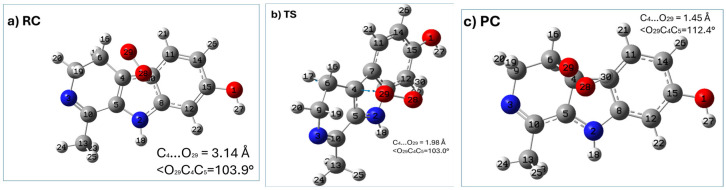
Graphical representations of the stationary points: (**a**) reactant complex (RC), (**b**) transition state (TS) and (**c**) product complex (PC) encountered along the RAF reaction pathway of harmalol (neutral form) with hydroperoxyl radicals. Displacement vectors of imaginary frequencies at 481.71*i* cm^−1^ are shown as blue arrows. Atomic labels according to Gaussian 09.

**Figure 5:**
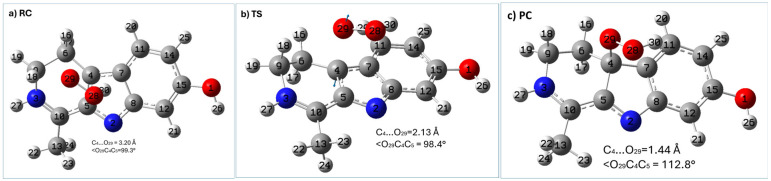
Graphical representations of the stationary points: (**a**) reactant complex (RC), (**b**) transition state (TS) and (**c**) product complex (PC) encountered along the RAF reaction pathway of harmalol (zwitterion I) with hydroperoxyl radicals. Displacement vectors of imaginary frequencies at 380.48*i* cm^−1^ are shown as blue arrows. Atomic labels according to Gaussian 09.

**Figure 6:**
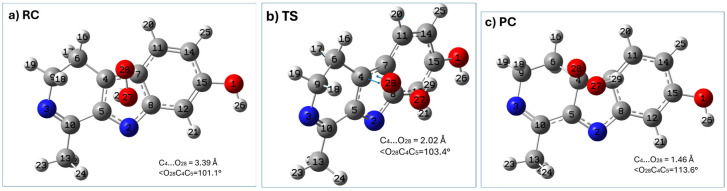
Graphical representations of the stationary points: (**a**) reactant complex (RC), (**b**) transition state (TS) and (**c**) product complex (PC) encountered along the RAF reaction pathway of harmalol (monoanion I) with hydroperoxyl radicals. Displacement vectors of imaginary frequencies at 243.47*i* cm^−1^ are shown as blue arrows. Atomic labels according to Gaussian 09.

**Figure 7:**
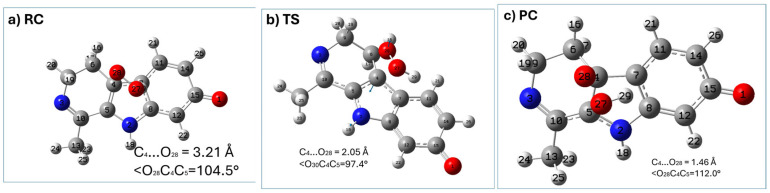
Graphical representations of the stationary points: (**a**) reactant complex (RC), (**b**) transition state (TS) and (**c**) product complex (PC) encountered along the RAF reaction pathway of harmalol (monoanion II) with hydroperoxyl radicals. Displacement vectors of imaginary frequencies at 367.54*i* cm^−1^ are shown as blue arrows. Atomic labels according to Gaussian 09.

**Figure 8:**
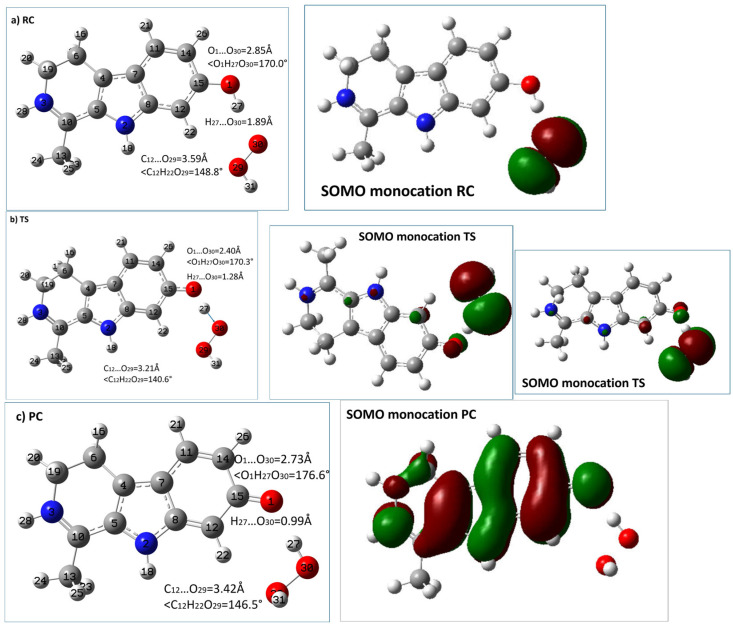
Graphical representations of the stationary points: (**a**) reactant complex (RC), (**b**) transition state (TS) and (**c**) product complex (PC) encountered along the FHT reaction pathway of harmalol (monocation) with hydroperoxyl radicals (left panel). Displacement vectors of imaginary frequencies at 2001*i* cm^−1^ are shown as blue arrows. Visualization of the SOMO distribution of the corresponding stationary points (right panel, isovalue 0.02). Atomic labels according to Gaussian 09.

**Figure 9:**
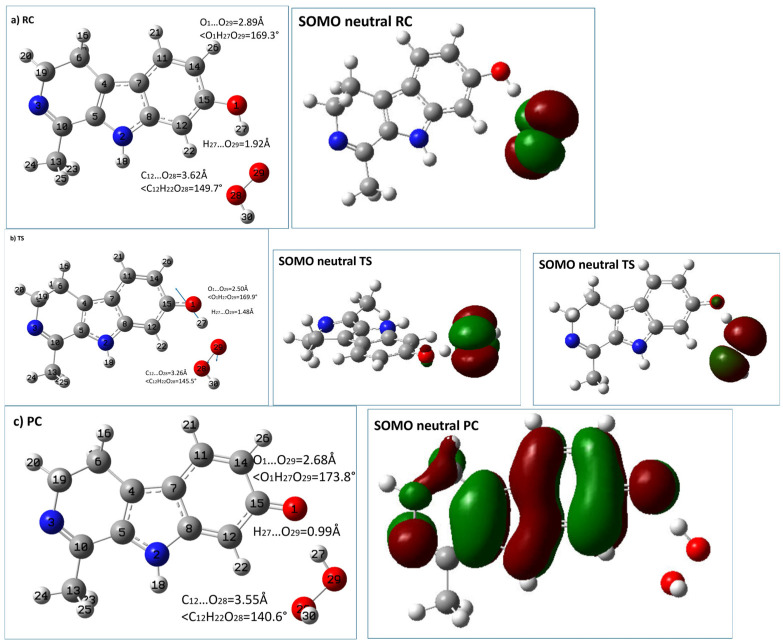
Graphical representations of the stationary points: (**a**) reactant complex (RC), (**b**) transition state (TS) and (**c**) product complex (PC) encountered along the FHT reaction pathway of harmalol (neutral form) with hydroperoxyl radicals (left panel). Displacement vectors of imaginary frequencies at 941*i* cm^−1^ are shown as blue arrows. Visualization of the SOMO distribution of the corresponding stationary points (right panel, isovalue 0.02). Atomic labels according to Gaussian 09.

**Figure 10:**
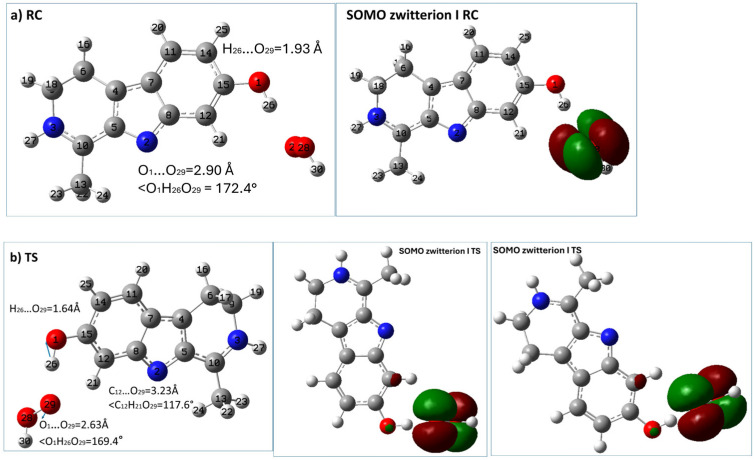

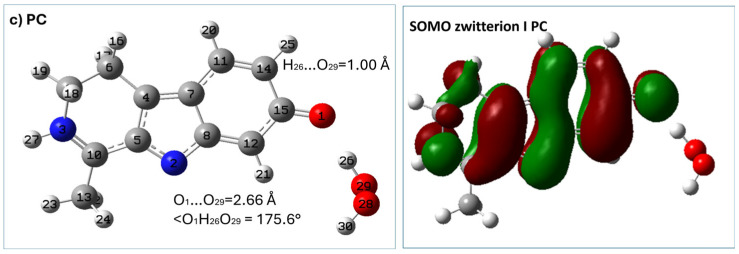
Graphical representations of the stationary points: (**a**) reactant complex (RC), (**b**) transition state (TS) and (**c**) product complex (PC) encountered along the FHT reaction pathway of harmalol (zwitterionic I form) with hydroperoxyl radicals (left panel). Displacement vectors of imaginary frequencies at 448.99*i* cm^−1^ are shown as blue arrows. Visualization of the SOMO distribution of the corresponding stationary points (right panel, isovalue 0.02). Atomic labels according to Gaussian 09.

Graphical representations of the stationary points (reactant complex (RC), transition state (TS) and product complex (PC)) along the reaction pathway within RAF mechanism of monocationic, neutral, zwitterion I and monoanionic (monoanion I and II) forms of harmalol with HOO^●^ radicals are presented in Figure 3, Figure 4, Figure 5, Figure 6 and Figure 7, whereas those encountered in the FHT mechanism for the monoanionic, neutral and zwitterionic I forms are shown in Figure 8, Figure 9 and Figure 10 together with the SOMO distribution of the corresponding stationary points. Kinetic parameters of the reactions involved in FHT and RAF antioxidant mechanisms are summarized in Table 6, together with Wigner transmission coefficient (κ) accounting for one-dimensional quantum tunneling effect. The kinetic data for SET reactions involving the monoanionic II form of harmalol and various radicals are summarized in Table 7. Values given in parentheses correspond to the SET reaction of monoanion I with the hydroperoxyl radical (HOO^•^).

As shown in Table 6, the endergonic RAF reactions involving the monocationic and neutral forms of harmalol proceed at very low rates, with apparent rate constants (k_app_) of 1.1 × 10^1^ and 1.3 × 10^2^ M^−1^ s^−1^, respectively. These rate constants indicate that the RAF pathway is kinetically unfavorable for both species and is therefore unlikely to contribute significantly to their radical-scavenging activity.

In contrast, the RAF reactions of zwitterion I, monoanion I, and monoanion II are considerably faster, with k_app_ values of 3.6 × 10^5^, 3.0 × 10^7^, and 1.4 × 10^5^ M^−1^ s^−1^, respectively. These findings indicate that the RAF mechanism may contribute to the radical-scavenging activity of these species, particularly in the case of monoanion I, for which the highest RAF rate constant was predicted. As also shown in Table 6, quantum tunneling has a negligible effect on the kinetics of the RAF reactions involving harmalol.

Inspection of the geometrical parameters of the stationary points along the RAF reaction pathways involving the monocationic, neutral, zwitterionic I, and monoanionic forms of harmalol (Figure 3, Figure 4, Figure 5, Figure 6 and Figure 7 and Tables S5–S9 in the Supplementary Material) reveals that the HOO^•^ radical initially approaches the C_4_ atom at a C_4_···O_28_(O_29_,O_30_) distance of ~3 Å and an O_28_(O_29_,O_30_)–C_4_–C_5_ angle of ~100°. Formation of the C_4_–O_28_(O_29_,O_30_) bond proceeds through a transition state characterized by a single imaginary frequency of 610.58*i* cm^−1^ (monocation), 481.71*i* cm^−1^ (neutral), 380.48*i* cm^−1^ (zwitterion I), 243.47*i* cm^−1^ (monoanion I), and 367.54*i* cm^−1^ (monoanion II), corresponding to the stretching motion along the forming C_4_–O_28_(O_29_,O_30_) bond. In the transition state, the C_4_···O_28_(O_29_,O_30_) distance decreases to ~2 Å, while the C_5_–C_4_–O_28_(O_29_,O_30_) angle remains close to ~100°. The transition state subsequently evolves into the product complex, in which the C_4_–O_28_(O_29_,O_30_) bond is fully formed (~1.45 Å) and the C_5_–C_4_–O_28_(O_29_,O_30_) angle reaches ~112°.

Kinetic parameters of the SET reactions involving the monoanionic II form of harmalol and a set of free radicals (Table 7) reveal very low activation barriers, ranging from 0.15 kcal mol^−1^ for highly reactive hydroxyl radicals to 4.97 kcal mol^−1^ for HOO^•^ radicals. Consequently, these reactions proceed rapidly, with bimolecular rate constants on the order of 10^9^–10^12^ M^−1^ s^−1^, indicating a kinetically favorable electron-transfer process. This behavior is consistent with Marcus theory, as the reactions occur outside the inverted region, with the absolute values of the Gibbs free energy changes (ΔG) remaining lower than the corresponding reorganization energies (λ) (Table 7) [5,11,32].

The calculated diffusion rate constants are on the order of 10^9^ M^−1^ s^−1^. For highly reactive radicals, whose intrinsic electron-transfer rate constants exceed the diffusion limit, the apparent rate constants are partially controlled by diffusion and approach the corresponding diffusion-controlled values. Nevertheless, the resulting apparent rate constants for SET reactions involving the monoanionic II form remain very high, typically on the order of 10^9^ M^−1^ s^−1^, indicating that the SET mechanism constitutes an efficient radical-scavenging pathway for this species.

In contrast, the corresponding SET reaction of monoanion I with the hydroperoxyl radical is approximately two orders of magnitude slower, with an apparent rate constant on the order of 10^6^ M^−1^ s^−1^, highlighting the strong influence of charge localization on the electron-donating ability of harmalol monoanions.

As shown in Table 5, the FHT reactions of the studied harmalol species are exergonic, with those involving the zwitterionic I and monoanionic forms being thermodynamically the most favorable. However, attempts to locate a transition state (TS) for the monoanionic forms were unsuccessful, as all transition state searches converged directly to the product structure rather than to a first-order saddle point. Various initial guesses and computational approaches were tested, yet all attempts led to the same outcome.

**Figure 11:**
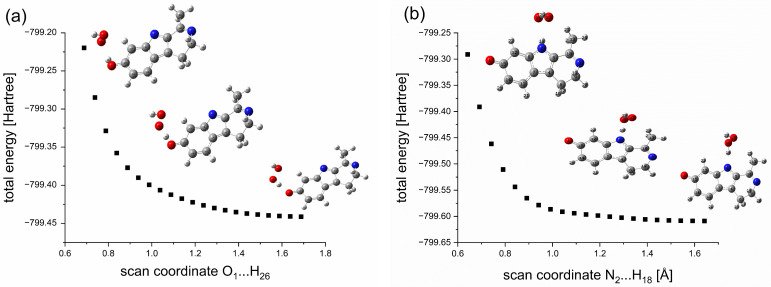
Relaxed potential energy surface scans showing the variation of the total energy along the scan coordinate corresponding to the O_1_…H_26_ distance in monoanion I and the N_2_…H_18_ distance in monoanion II during hydrogen transfer between (**a**) monoanionic form I and (**b**) monoanionic form II of harmalol and the hydroperoxyl radical. Atomic labeling follows the Gaussian 09 convention.

To further investigate the reaction profile, a relaxed potential energy surface scan was performed by varying the O_1_–H_26_/N_2_–H_18_ interatomic distance associated with the hydrogen-transfer process (see Figure 11).

The results reveal a continuous, monotonic decrease in energy from the reactant complex toward the product, with no detectable maximum along the reaction path. This behavior indicates that the reaction proceeds without a discernible activation barrier and can be considered effectively barrierless. Consequently, hydrogen transfer is expected to be highly efficient and may represent an important radical-scavenging pathway under alkaline conditions.

In contrast, FHT reactions involving the neutral, zwitterionic I and monocationic forms of harmalol proceed via well-defined transition states (see Figure 8, Figure 9 and Figure 10). All stationary points along the reaction pathway involving the monocationic and neutral forms are characterized by the presence of intermolecular hydrogen bonding interactions. In the reactant complex (RC), a hydrogen bond is formed between the O_1_H_27_ group of harmalol and the O_30_ (or O_29_) atom of the HOO^•^ radical, whereas in the product complex (PC), the interaction occurs between the OH group of hydrogen peroxide and the O_1_ atom of harmalol. The geometrical parameters indicate that these interactions are strong, as evidenced by nearly linear arrangements and O…O distances below 3 Å (see Figure 8 and Figure 9).

In particular, in the transition state the O_1_…O_30_(O_29_) distance is approximately 2.50 Å, and the O_1_H_27_O_30_(O_29_) angle is about 170°, confirming the strength of the hydrogen bond. The FHT process proceeds through a transition state characterized by a single imaginary frequency of 2001*i* cm^−1^ and 941*i* cm^−1^ for the monocationic and neutral forms, respectively, corresponding to the stretching vibration of the O_1_H_27_ bond. During the reaction, the hydrogen atom is transferred to the radical, as reflected by the change in the H_27_O_30_(O_29_) distance from about 1.90 Å in the RC to 1.28–1.48 Å in the TS and finally to 0.99 Å in the PC (see Figure 8 and Figure 9).

The reactant complex of zwitterion I (Figure 10) is stabilized by a strong (linear) hydrogen bond formed between O_1_H_26_ and the oxygen atom (O_29_) of hydroperoxyl radical (O_1_O_29_ = 2.90 Å). This interaction is preserved in the transition state, where the O_1_O_29_ distance is approximately 2.60 Å and the O_1_H_26_O_29_ angle remains close to linearity (ca. 170°). The reaction proceeds through a transition state characterized by a single imaginary frequency of 449*i* cm^−1^ corresponding to the stretching vibration of the O_1_–H_26_ bond. During the hydrogen-transfer process, the H_26_O_29_ distance decreases from about 1.90 Å in the reactant complex to 1.64 Å in the transition state and finally to 1.00 Å in the product complex.

As shown in Table 6, the formal hydrogen transfer (FHT) reaction proceeds approximately three orders of magnitude faster for the neutral form of harmalol (k_app_ = 5.0 × 10^8^ M^−1^ s^−1^) than for its monocationic counterpart (k_app_ = 5.5 × 10^5^ M^−1^ s^−1^). An even greater enhancement is observed for zwitterion I, for which the calculated rate constant (k_app_ = 2.1 × 10^9^ M^−1^ s^−1^) is approximately 4 orders of magnitude higher than that of the monocationic species. This trend is consistent with the thermodynamic analysis, which indicates increasingly favorable (more exergonic) hydrogen-transfer reactions for the neutral and zwitterionic forms. However, for monocationic species a higher Wigner tunneling correction factor (κ = 4.9) is obtained compared to the neutral species (κ = 1.9) and zwitterionic I (κ = 1.1) species. This difference can be attributed to the significantly larger imaginary frequency of the transition state for the monocation (2001*i* cm^−1^ vs. 941*i* cm^−1^ (neutral form) and 449*i* cm^−1^ (zwitterion I)), which reflects a narrower potential energy barrier and thus enhances quantum tunneling. Nevertheless, tunneling provides only a minor contribution to the overall kinetics of the FHT reaction, and the reaction rate is primarily governed by the activation free energy.

The analysis of the singly occupied molecular orbital (SOMO) distribution along the FHT reaction pathway (RC → TS → PC) may provide deeper insight into the nature of the hydrogen transfer mechanism, i.e., whether it proceeds via a hydrogen atom transfer (HAT) or a proton-coupled electron transfer (PCET) pathway [11,33]. As shown in Figure 8, Figure 9 and Figure 10 (right panels), in the reactant complex (RC), the SOMO is localized exclusively on the HOO^•^ radical, indicating that the unpaired electron is initially confined to the radical species.

As the system evolves toward the transition state (TS), the SOMO remains primarily centered on the radical but shows partial delocalization onto the O_1_ atom of harmalol. Notably, no significant SOMO density is observed on the transferring hydrogen atom, suggesting that the electron and proton are not transferred as a single hydrogen atom. In the product complex (PC), the SOMO becomes fully localized on the harmalol moiety, indicating completion of the electron transfer process.

This evolution of the SOMO distribution suggests that the reaction does not proceed via a simple HAT mechanism, but rather involves a proton-coupled electron transfer (PCET) pathway, in which electron and proton transfer occur in a concerted but not strictly synchronous manner.

It is generally accepted [11,33] that, for HAT reactions, the SOMO in the transition state is distributed along the hydrogen-transfer coordinate between the donor and acceptor, whereas for PCET it is oriented orthogonally to this axis. As shown in Figure 8, Figure 9 and Figure 10, the TS SOMO is dominated by orbitals of predominant p character perpendicular to the donor-H-acceptor transition vector, which signifies that the electron and proton are transferred through different orbital channels (different sets of orbitals). This behavior is consistent with a decoupled electron–proton transfer picture typical of PCET processes. To further elucidate the nature of this mechanism, additional analyses of spin density and atomic charges along the reaction coordinate were performed, as discussed in the following section.

### 2.5. Evidence for a PCET Mechanism

The evolution of spin density distribution and natural atomic charges along the intrinsic reaction coordinate (IRC) was analyzed for key atoms of the monocationic, neutral and zwitterionic I forms of harmalol involved in the FHT reaction. Complete numerical data for all atoms along the IRC for the neutral and monocationic forms are provided in the Supplementary Information (Tables S10–S13).

**Figure 12:**
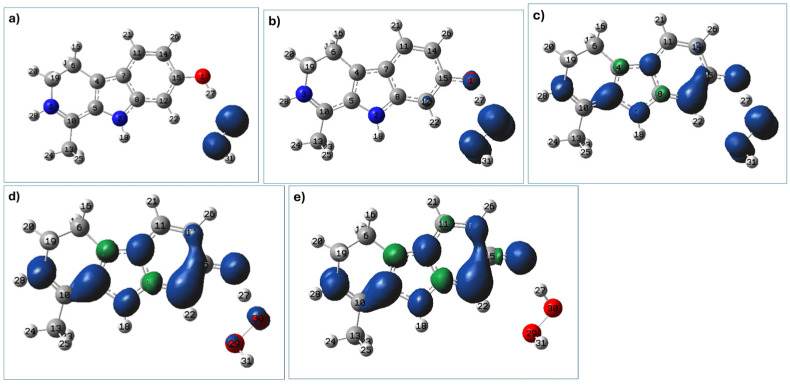
Spin density distribution along the intrinsic reaction coordinate (IRC) for the formal hydrogen transfer (FHT) mechanism in monocationic harmalol: (**a**) reactant complex (RC), (**b**) pre-transition state (pre-TS), (**c**) transition state (TS), (**d**) post-transition state (post-TS), and (**e**) product complex (PC). Spin density isosurfaces were plotted at an isovalue of 0.0025 a.u.

**Figure 13:**
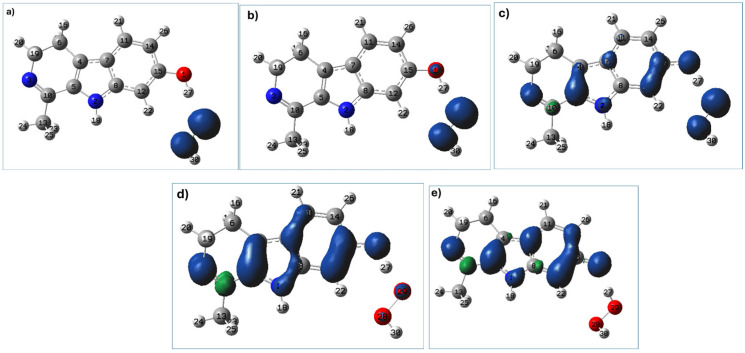
Spin density distribution along the intrinsic reaction coordinate (IRC) for the formal hydrogen transfer (FHT) mechanism in neutral harmalol: (**a**) reactant complex (RC), (**b**) pre-transition state (pre-TS), (**c**) transition state (TS), (**d**) post-transition state (post-TS), and (**e**) product complex (PC). Spin density isosurfaces were plotted at an isovalue of 0.0025 a.u.

**Figure 14:**
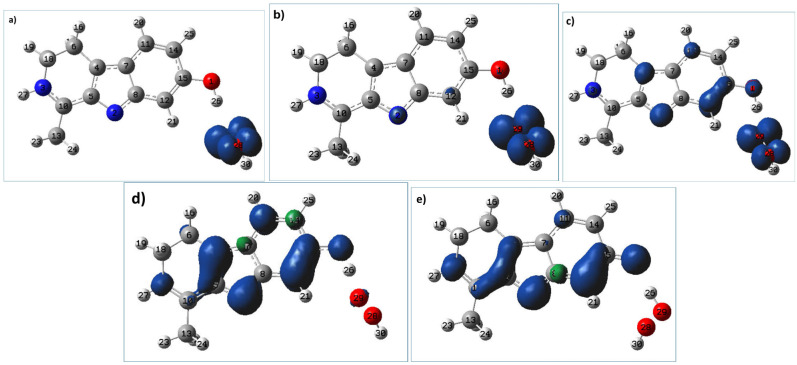
Spin density distribution along the intrinsic reaction coordinate (IRC) for the formal hydrogen transfer (FHT) mechanism in zwitterionic I form of harmalol: (**a**) reactant complex (RC), (**b**) pre-transition state (pre-TS), (**c**) transition state (TS), (**d**) post-transition state (post-TS), and (**e**) product complex (PC). Spin density isosurfaces were plotted at an isovalue of 0.0025 a.u.

The spin density evolution along the IRC for monocationic, neutral, zwitterionic I forms is presented in Figure 12, Figure 13 and Figure 14, showing the progression from the reactant complex (RC), through the pre-transition state (pre-TS) and transition state (TS), to the post-transition state (post-TS) and product complex (PC). The corresponding changes in natural charges and spin densities for the monocationic, neutral and zwitterionic I forms are summarized in Table 8.

As shown in Table 8, in the monocationic, neutral and zwitterionic I forms of harmalol, the spin density on the transferring hydrogen atom (H_27_/H_26_) remains negligible (<0.005) throughout the reaction path and approaches zero in the transition state region. This clearly excludes a hydrogen atom transfer (HAT) mechanism, in which significant spin density would be expected on the hydrogen atom. Instead, unpaired electron is initially localized on the oxygen atoms of the donor–acceptor moiety and subsequently redistributes over the π-system of the molecule as the reaction proceeds, indicating that electron transfer is not localized on the proton (Figure 12, Figure 13 and Figure 14). Concurrently, the natural charge analysis reveals a continuous, though not strictly monotonic, redistribution of electron density between the donor and acceptor sites, with almost unchanged charge on transferring proton H_27/26_, confirming its proton-like (H^+^) character. The observed gradual redistribution of charge between donor and acceptor sites, together with the absence of spin density on hydrogen, supports a PCET mechanism.

## 3. Materials and Methods

### 3.1. Electronic and Geometrical Structures

Geometry optimizations and subsequent frequency calculations of H_2_O_2_ and harmalol (in its monocationic, neutral, zwitterionic and monoanionic forms), as well as the corresponding radical species, were performed at the M06-2X/6-311+G(d,p)/PCM(water) level of theory. This level of theory was selected as a compromise between computational accuracy and cost, while ensuring a reliable description of radical and ionic species through the inclusion of diffuse and polarization functions. Unrestricted calculations were applied to open-shell species (radicals). Frequency analyses were used to confirm the nature of the obtained stationary points: no imaginary frequencies were found for local minima, while transition states were characterized by a single imaginary frequency.

Atomic charges and spin densities were obtained using Natural Population Analysis (NPA) within the Natural Bond Orbital (NBO) framework, as well as the Hirshfeld partitioning scheme. All calculations were carried out using Gaussian 09 [34] and GaussView 5.0 [35].

### 3.2. Deprotonation Constants

pKa values corresponding to each of the deprotonation steps (pKa_1_, pKa_2_) of harmalol were calculated at the M06-2X/6-311+G(d,p)/PCM(water) level of theory using the previously reported [29] parameter-fitting methodology.

### 3.3. Global Reactivity Descriptors

Global reactivity descriptors of harmalol were obtained at the M06-2X/6-311+G(d,p)/PCM (water) level of theory. The ionization potential (IP) and electron affinity (EA) were approximated using Koopmans’ theorem [36] as the negative energies of the HOMO and LUMO orbitals, respectively. Using these values, electronegativity (χ), chemical potential (μ), global hardness (η), global softness (S), electrophilicity index (ω), as well as electron-donating (ω^−^) and electron-accepting (ω^+^) powers were determined [9,37,38,39].

### 3.4. Intrinsic Thermochemical Reactivity Indices

Intrinsic thermochemical reactivity indices, including bond dissociation enthalpy (BDE), adiabatic ionization potential (AIP), proton affinity (PA), electron transfer enthalpy (ETE) and proton dissociation enthalpy (PDE) were calculated at the M06-2X/6-311+G(d,p)/PCM(water) level of theory using the previously described methodology [9,40]. The enthalpies of solvated proton (−1052.7 kJ/mol) and electron (−98.8 kJ/mol) were taken from [41].

### 3.5. Thermodynamics of HOO^•^ Radical Scavenging Pathways

The Gibbs free energies of reactions associated with FHT (formal hydrogen transfer), RAF (radical adduct formation), SET (single electron transfer), SET-PT (sequential electron transfer–proton transfer), SPL-ET (sequential proton loss–electron transfer), and SPL-FHT (sequential proton loss–formal hydrogen transfer) were calculated at the M06-2X/6-311+G(d,p)/PCM (water) level of theory using the methodology described previously [5,9,40]. For RAF processes, due to their bimolecular character (2 → 1), the Gibbs free energies were corrected to the 1 M standard state.

### 3.6. Rate Constants for FHT and RAF Reactions

In order to find and optimize transition state structures in FHT and RAF reaction pathways, the method implemented by Schlegel and coworkers [42,43] was applied (requested by the QST3 option in Gaussian 09). Then, for the identified TS, the intrinsic reaction coordinate (IRC) was constructed starting from the respective TS geometry and going downhill to both the reactant and the product channels to ensure that the obtained TS corresponds to the reactions involved in FHT and RAF mechanisms.

However, for the monoanionic forms of harmalol, no valid transition state in the FHT pathway was identified. Despite extensive efforts, all FHT transition state searches consistently converged to the product structure rather than to a first-order saddle point. Various approaches and initial guesses were tested; however, all attempts led to the same outcome. Therefore, a relaxed potential energy surface (PES) scan was performed at the UM06-2X/6-311+G(d,p)/PCM(water) level by varying the O_1_…H_26_ (monoanion I) and N_2_…H_18_ (monoanion II) distance in the product complex (PC) formed between the monoanionic form of harmalol and the HOO^•^ radical. The N_2_–H_18_/O_1_…H_26_ distance was changed in 20 steps with a decrement of 0.05 Å, while all remaining geometric parameters were fully optimized at each step.

The bimolecular rate constants for reactions in the FHT and RAF pathways were calculated at the M06-2X/6-311+G(d,p)/PCM(water) level within the framework of conventional transition state theory (TST), including tunneling corrections and reaction path degeneracy, and using a standard state of 1 M, according to the following equation [5,8,9,44,45]:

$$k_{bim}=\sigma \kappa (T)\frac{k_{B}T}{h}e^{-(∆G_{a}^{‡})/RT}$$

The tunneling correction factor κ(T) was evaluated using the Wigner approximation [46]:

$${ĸ\left(T\right)=1+\frac{1}{24}[\frac{h\left|im\left(\nu^{‡}\right)\right|}{k_{B}T}]}^{2}$$

where: σ is the reaction path degeneracy (i.e., the number of equivalent reaction pathways) [47,48], κ(T) is the Wigner tunneling correction factor, ν^‡^ is the imaginary vibrational frequency of the transition state, and im(ν^‡^) denotes its magnitude corresponding to motion along the reaction coordinate; k_B_ is the Boltzmann constant, h is the Planck constant, T is the standard temperature, R is the gas constant, and $∆G_{a}^{‡}$ is the Gibbs free energy of activation. All rate constants were calculated using the Eyringpy program [49,50].

### 3.7. Rate Constant for Single Electron Transfer (SET) Reaction

In the SET mechanism, no transition state (TS) is present between reactants and products. The activation free energy for electron transfer was calculated based on Marcus’s theory [51,52], according to the following equation:

$${\Delta G}_{a}^{‡}=\frac{\lambda }{4}{\left(1+\frac{∆G}{\lambda }\right)}^{2}$$

where λ is the nuclear reorganization energy, calculated as the difference between ΔE (the vertical energy difference between reactants and products in the SET process) and the adiabatic free energy of reaction ΔG:

λ=∆E−∆G

The bimolecular rate constant for the SET reaction (k_bim_) was calculated using the following equation [44]:

$$k_{bim}=\frac{k_{B}T}{h}exp\left(\frac{-{\Delta G}_{a}^{‡}}{RT}\right)$$

where k_B_—Boltzmann constant, h—Planck constant, R—gas constant, T—absolute temperature.

### 3.8. Correction for Diffusion-Controlled Rates

For bimolecular rate constants approaching the diffusion limit, the apparent rate constants (k_app_) were calculated using the Collins–Kimball theory [53]:

$$k_{app}=\frac{k_{D}k_{bim}}{k_{D}+k_{bim}}$$

where k_D_ is the rate constant for an irreversible bimolecular diffusion-controlled reaction according to Smoluchowski [54]:

$$k_{D}=4\pi R_{AB}D_{AB}N_{A}$$

where N_A_ is the Avogadro number; R_AB_ is the reaction distance (taken as the sum of the reactants’ radii); D_AB_ is the mutual diffusion coefficient of the antioxidant A and radical B. It is calculated as the sum of individual diffusion coefficients obtained from the Stokes–Einstein equation [55,56]:

$$D_{A}=\frac{k_{B}T}{{6\pi \eta R}_{A}}$$

$$D_{B}=\frac{k_{B}T}{{6\pi \eta R}_{B}}$$

where η is the viscosity of water (8.905 × 10^−4^ Pa·s [57]).

## 4. Conclusions

The results of the M06-2X/6-311+G(d,p)/PCM(water) calculations performed in this study demonstrate that the antioxidant activity of harmalol is strongly pH-dependent and closely related to its protonation state and tautomeric form. Under highly acidic conditions (pH 2), harmalol exists exclusively in the monocationic form, whereas at physiological pH (7.4) the monocation remains the predominant species (92.64%), accompanied by a smaller fraction of the neutral and zwitterionic forms. In contrast, under strongly basic conditions (pH 13), the monoanionic forms become dominant (98.44%). The neutral species, accompanied by zwitterionic tautomers, prevails mainly within the pH range between pKa_1_ and pKa_2_. Consequently, the relative importance of the individual radical-scavenging mechanisms changes with pH owing to the distinct reactivities and abundances of the various protonation states and tautomeric species.

For the monocationic, neutral and zwitterionic I forms, formal hydrogen transfer (FHT) reactions proceed through well-defined transition states and are governed primarily by the activation free energy, whereas tunneling effects contribute only marginally to the overall reaction kinetics. Detailed analyses of SOMO distributions, spin density evolution, and natural atomic charges along the intrinsic reaction coordinate clearly indicate that FHT reactions involving these species of harmalol do not follow a classical hydrogen atom transfer (HAT) pathway. Instead, the mechanism proceeds via proton-coupled electron transfer (PCET), characterized by coupled but asynchronous proton and electron transfer. This conclusion is supported by the negligible spin density on the transferring hydrogen atom and by the orientation of the SOMO perpendicular to the proton-transfer coordinate in the transition state.

The zwitterionic I and neutral forms exhibit significantly higher apparent rate constants for the PCET reaction (k_app_ = 2.1 × 10^9^ M^−1^ s^−1^, k_app_ = 5.0 × 10^8^ M^−1^ s^−1^, respectively) than the monocationic form (k_app_ = 5.5 × 10^5^ M^−1^ s^−1^). Additionally, zwitterionic I forms contribute to the scavenging of HOO radicals via RAF mechanism with k_app_ = 3.6×10^5^ M^−1^ s^−1^. In contrast, the RAF pathway is kinetically negligible for the monocationic and neutral forms.

The results of the calculations indicate that monoanion II exhibits a high electron-donating ability, favoring rapid single-electron transfer (SET) reactions toward hydroperoxyl radicals and potentially toward other electrophilic radical species. Kinetic parameters of the SET reactions involving monoanion II and various free radicals reveal very low activation barriers. Consequently, these reactions proceed rapidly, with bimolecular rate constants on the order of 10^9^–10^12^ M^−1^ s^−1^, indicating a kinetically favorable electron-transfer process. Although diffusion partially limits the overall reaction rate for the most reactive radicals, the resulting apparent rate constants remain very high, typically on the order of 10^9^ M^−1^ s^−1^. In contrast, the SET reactivity of monoanion I is approximately two orders of magnitude lower than that of monoanion II, highlighting the strong influence of charge localization on the electron-donating ability of harmalol monoanions. Overall, these findings indicate that the SET mechanism constitutes an efficient radical-scavenging pathway for monoanion II and may also contribute to the antioxidant activity of monoanion I.

Additionally, for both monoanionic forms, FHT reactions are thermodynamically favorable. The inability to locate a transition state for these monoanionic forms, together with the monotonic decrease in energy along the scanned reaction coordinate, indicates a barrierless hydrogen-transfer process. Consequently, hydrogen transfer is expected to be highly efficient and may represent an important antioxidant pathway under alkaline conditions.

Moreover, thermodynamic and kinetic analyses revealed that the radical adduct formation (RAF) mechanism may also contribute to the antioxidant activity of the monoanionic forms of harmalol. In particular, RAF reactions involving monoanion I and monoanion II proceed with apparent rate constants of 3.0 × 10^7^ and 1.4 × 10^5^ M^−1^ s^−1^, respectively. These results indicate that RAF may become a competitive radical-scavenging pathway, especially for monoanion I under strongly alkaline conditions.

The calculated rate constants for individual antioxidant mechanisms represent the intrinsic reactivity of the corresponding acid–base species of harmalol. It should be emphasized that a meaningful comparison with experimental data requires accounting for the pH-dependent distribution of harmalol species as well as the HOO^•^/O_2_^−•^ acid–base equilibrium. Accordingly, the apparent rate constants should be weighted by the molar fractions of the reactive species present under the considered conditions.

Overall, the present theoretical study provides detailed insight into the pH-dependent antioxidant behavior of harmalol and demonstrates that its radical-scavenging efficiency increases with increasing pH.

Under physiological conditions, the monocationic form predominates, with a smaller contribution from the neutral/zwitterionic I and II species, and radical scavenging proceeds mainly via proton-coupled electron transfer (PCET)-type hydrogen-transfer reactions involving monocationic, neutral and zwitterionic I forms as well as the RAF mechanism involving zwitterion I. In contrast, under alkaline conditions the monoanionic species predominate and react through highly favorable FHT pathways, accompanied by efficient SET and RAF mechanisms. These findings not only clarify the antioxidant mechanism of harmalol but also broaden the understanding of free-radical scavenging processes in structurally related β-carboline and phenolic compounds. The identified thermodynamic and kinetic preferences provide valuable insight into the molecular basis of the antioxidant activity of harmalol and may assist in the rational design of novel antioxidant compounds based on the β-carboline scaffold.

Future studies will focus on the investigation of harmalol antioxidant mechanisms in nonpolar environments, as well as on reactions involving additional reactive oxygen and nitrogen species. Experimental validation of the theoretical predictions reported herein would further strengthen the understanding of the biological relevance of harmalol as a natural antioxidant.

## Figures and Tables

**Table 1:** M06-2X/6-311+G(d,p)/PCM(water) values of pKa for harmalol determined with the use of parameter fitting method.

pKa	Parameter Fitting Method	Experimental Literature Value
pKa_1_	8.4	8.5 [22,26], 8.6 [25]
pKa_2_	10.8	11.2 [26], 11.3 [22], 11.5 [25]

**Table 2:** Relative Gibbs free energies of harmalol species involved in the neutral–zwitterionic and monoanionic tautomeric equilibria, calculated at the M06-2X/6-311+G(d,p)/PCM (water) level.

Species	Relative Gibbs Free Energy (kcal mol^−1^)
neutral/zwitterionic equilibrium	
neutral	0.000
zwitterion I	6.488
zwitterion II	5.580
monoanionic equilibrium	
monoanion I	3.032
monoanion II	0.000

**Table 3:** M06-2X/6-311+G(d,p)/PCM(water) calculated global reactivity descriptors.

Harmalol	IP [kcal/mol]	EA [kcal/mol]	χ [kcal/mol]	μ [kcal/mol]	η [kcal/mol]	S [kcal/mol]	ω [kcal/mol]	ω− [kcal/mol]	ω+ [kcal/mol]
monocation	173.72	44.43	109.08	−109.08	64.64	32.32	92.03	154.65	23.01
neutral	157.79	12.19	84.99	−84.99	72.80	36.40	49.61	101.20	12.40
zwitterion I	149.32	27.77	88.54	−88.54	60.77	30.39	64.50	116.37	16.12
zwitterion II	141.95	33.40	87.67	−87.67	54.28	27.14	70.81	121.43	17.70
monoanion I	134.07	−1.79	66.14	−66.14	67.93	33.97	32.20	73.76	8.05
monoanion II	128.06	2.22	65.14	−65.14	62.92	31.46	33.72	74.15	8.43

**Table 4:** M06-2X/6-311+G(d,p)/PCM(water) calculated values of adiabatic IP (AIP), BDE, PA, ETE and PDE.

Harmalol	AIP [kcal/mol]	BDE [kcal/mol]	PA [kcal/mol]	ETE [kcal/mol]	PDE [kcal/mol]
monocation (MC)	121.59	86.14 (O_1_H) 124.91 (N_2_H)	26.18 (MC→N)	-	-
neutral (N)	105.39	80.60 (O_1_H) 87.78 (N_2_H)	40.52 (N→MA II)	-	11.11
zwitterion I	95.49	75.98 (O_1_H)	-	-	-
zwitterion II	90.18	77.01 (N_2_H)	-	-	-
monoanion I	80.17	70.91 (O_1_H)	-	70.91	-
monoanion (MA II)	75.98	73.89 (N_2_H)	-	73.90	-

AIP—adiabatic ionization potential, BDE—bond dissociation energy, PA—proton affinity, ETE—electron transfer enthalpy, PDE—proton dissociation enthalpy.

**Table 5:** M06-2X/6-311+G(d,p)/PCM(water) Gibbs free energies [kcal/mol] of the reactions involved in the FHT, SET, SET-PT, SPL-ET, and SPL-FHT antioxidant mechanisms of harmalol towards HOO^●^ radicals.

Harmalol	RAF ∆G [kcal/mol]	FHT ∆G [kcal/mol]	SET ∆G [kcal/mol]	SET-PT ∆G [kcal/mol]	SPL-FHT ∆G [kcal/mol]	SPL-ET ∆G [kcal/mol]
monocation	−1.913 (C_4_)	−2.975 (O_1_)	+49.955	(1) +49.955 (2) −50.476 (O_1_)	(1) * −35.673 (2) −9.372	(1) * −35.673 (2) +33.536
neutral	+1.477 (C_4_)	−9.372 (O_1_)	+33.536	(1) +33.534 (2) −39.534 (O_1_)	(1) ** −21.069 (2) −12.607	(1) ** −21.069 (2) +4.014
zwitterion I	−11.042 (C_4_)	−12.966 (O_1_)	+23.32	-	-	-
zwitterion II	+0.015 (C_4_)	−10.409 (N_2_)	+18.510	-	-	-
monoanion I	−2.000 (C_4_)	−15.641 (O_1_)	+8.077	-	-	-
monoanion II	−1.596 (C_4_)	−12.607 (N_2_)	+4.014	-	-	-

Atomic labels according to Gaussian 09-see Figure 1. * SPL reaction corresponding to monocation + OH^−^→neutral + H_2_O; ** SPL reaction corresponding to neutral + OH^−^→monoanion II + H_2_O.

**Table 6:** Kinetic parameters (Gibbs free energies of activation (ΔG_a_ ^‡^), Wigner transmission coefficient (κ), diffusion rate constants (k_D_), bimolecular rate constant (k_bim_), and diffusion-corrected apparent rate constants (k_app_)) of the reactions involved in the FHT and RAF antioxidant mechanisms (for monocationic, neutral, zwitterionic I and monoanionic species of harmalol) towards HOO^●^.

Mechanism	Harmalol Species	ΔG_a_ ^‡^ [kcal/mol]	κ	k_D_ [M^−1^s^−1^]	k_bim_ [M^−1^s^−1^]	k_app_ [M^−1^s^−1^]
FHT	monocation	11.4	4.9	2.5 × 10^9^	5.5 × 10^5^	5.5 × 10^5^
FHT	neutral	6.6	1.9	2.6 × 10^9^	6.2 × 10^8^	5.0 × 10^8^
FHT	zwitterion I	4.8	1.2	2.7 × 10^9^	9.5 × 10^9^	2.1 × 10^9^
RAF	monocation	16.9	1.4	2.0 × 10^9^	1.0 × 10^1^	1.0 × 10^1^
RAF	neutral	15.3	1.2	2.0 × 10^9^	1.3 × 10^2^	1.3 × 10^2^
RAF	zwitterion I	10.6	1.1	2.2 × 10^9^	3.6 × 10^5^	3.6 × 10^5^
RAF	monoanion I	7.9	1.1	2.1 × 10^9^	3.0 × 10^7^	3.0 × 10^7^
RAF	monoanion II	11.2	1.1	2.0 × 10^9^	1.4 × 10^5^	1.4 × 10^5^

**Table 7:** Kinetic parameters (reaction Gibbs free energies (ΔG), Gibbs free energies of activation (ΔG_a_ ^‡^), reorganization energies (λ), rate constants (k_bim_), diffusion rate constants (k_D_), diffusion-corrected apparent rate constants (k_app_)) for SET reactions of the monoanionic II form of harmalol with various radicals. Values in parentheses correspond to the SET reaction involving monoanion I and the hydroperoxyl radical.

Radical	ΔG [kJ/mol]	ΔG_a_^‡^ [kJ/mol]	λ [kJ/mol]	k_bim_ [M^−1^s^−1^]	k_D_ [M^−1^s^−1^]	k_app_ [M^−1^s^−1^]
HOO^•^	16.793 (33.795)	20.785 (33.816)	42.994 (35.514)	1.42 × 10^9^ (7.39 × 10^6^)	7.95 × 10^9^ (7.81 × 10^9^)	1.20 × 10^9^ (7.38 × 10^6^)
NO_2_^•^	−84.470	15.173	192.581	1.36 × 10^10^	8.00 × 10^9^	5.05 × 10^9^
HO^•^	−70.912	0.624	85.519	4.83 × 10^12^	8.29 × 10^9^	8.27 × 10^9^
SO_4_^•−^	−158.570	6.623	237.968	4.29 × 10^11^	7.64 × 10^9^	7.50 × 10^9^
CH_3_O^•^	−9.785	4.711	35.733	9.29 × 10^11^	7.94 × 10^9^	7.87 × 10^9^
Cl_3_CO^•^	−280.574	6.639	381.190	4.27 × 10^11^	7.56 × 10^9^	7.43 × 10^9^

**Table 8:** Evolution of natural charges and spin density along the Intrinsic Reaction Coordinate for the key atoms involved in FHT mechanism for neutral, monocationic and zwitterionic I form of harmalol.

	**NEUTRAL FORM**					
		**RC**	**Pre-TS**	**TS**	**Post-TS**	**PC**
natural charge	O_1_	−0.35749	−0.35309	−0.28754	−0.23718	−0.21516
	H_27_	0.24577	0.23763	0.24312	0.24191	0.24451
	O_29_	0.26034	0.23785	−0.04813	−0.31864	−0.25735
spin density	O_1_	0.000932	0.012325	0.091485	0.145264	0.218258
	H_27_	0.002127	0.004048	−0.000014	0.002535	0.001982
	O_29_	0.672092	0.632919	0.332712	0.022849	0.000617
O_1_…H_27_ [Å]		0.97	0.96	1.03	1.10	1.69
H_27_…O_29_ [Å]		1.92	1.59	1.48	1.36	0.99
O_1_…O_29_ [Å]		2.89	2.55	2.50	2.46	2.68
	**MONOCATIONIC FORM**					
		**RC**	**Pre-TS**	**TS**	**Post-TS**	**PC**
natural charge	O_1_	−0.34786	−0.33677	−0.24756	−0.18477	−0.17716
	H_27_	0.24757	0.23370	0.23462	0.23208	0.24365
	O_30_	0.26136	0.22615	−0.05184	−0.27082	−0.25350
spin density	O_1_	0.000083	0.027758	0.130418	0.194152	0.232945
	H_27_	0.002461	0.005410	0.000753	0.002392	0.001568
	O_30_	0.671736	0.599338	0.292849	0.030800	0.000058
O_1_…H_27_ [Å]		0.98	1.02	1.12	1.23	1.74
H_27_…O_30_ [Å]		1.98	1.41	1.28	1.16	0.99
O_1_…O_30_ [Å]		2.85	2.42	2.40	2.38	1.73
		**ZWITTERION I**				
		**RC**	**Pre-TS**	**TS**	**Post-TS**	**PC**
natural charge	O_1_	−0.36007	−0.35275	−0.33391	−0.28136	−0.24821
	H_26_	0.24446	0.24674	0.24626	0.24542	0.24435
	O_29_	0.26136	0.21264	0.04242	−0.33753	−0.26035
spin density	O_1_	−0.000965	0.008739	0.035944	0.098795	0.178266
	H_26_	0.001830	0.000916	0.001367	0.002855	0.001489
	O_29_	0.670926	0.622019	0.439730	0.009541	−0.000452
O_1_…H_26_ [Å]		0.98	0.99	1.00	1.09	1.67
H_26_…O_29_ [Å]		1.90	1.87	1.64	1.41	1.00
O_1_…O_29_ [Å]		2.88	2.84	2.63	2.49	2.69

## Data Availability

The original contributions presented in this study are included in the article/Supplementary Material. Further inquiries can be directed to the corresponding author.

## Supplementary Materials

The following supporting information can be downloaded at: https://www.mdpi.com/article/10.3390/ijms27135959/s1.
