# Supplementary material for: pH-Dependent Antioxidant Mechanisms of Harmalol Toward HOO• Radicals in Aqueous Solution: A Quantum Chemical Study
Source: Int J Mol Sci. 2026 Jul 2;27(13):5959. doi: 10.3390/ijms27135959 (PMC13362251; doi:10.3390/ijms27135959)
Supplement: Supplementary file 1 [file ijms-27-05959-s001.zip › ijms-4341569-supplementary.pdf]

SUPPLEMENTARY MATERIAL

Table S1. M06-2X/6-311+G(d,p)/PCM(water) electronic energies [kcal/mol] of the reactions involved in the FHT, SET, SET-PT, SPL-ET, and SPL-FHT antioxidant mechanisms of harmalol towards HOO• radicals.

harmalol	RAF ΔE [kcal/mol]	FHT ΔE [kcal/mol]	SET ΔE [kcal/mol]	SET-PT ΔE [kcal/mol]	SPL-FHT ΔE [kcal/mol]	SPL-ET ΔE [kcal/mol]
monocation	-12.162 (C_4)	-3.006 (O_1)	+49.730	(1)+49.730<br>(2)-49.955 (O_1)	(1)*-34.587<br>(2)-9.440	(1)*-34.587<br>(2)+33.532
neutral	-8.446 (C_4)	-9.440 (O_1)	+33.532	(1)+33.532<br>(2)-39.297 (O_1)	(1)**-20.247<br>(2)-12.469	(1)** -20.247<br>(2)+4.116
zwitterion I	-21.258 (C_4)	-12.924 (O_1)	+23.633	-	-	-
zwitterion II	-10.414 (C_4)	-9.349 (N_2)	+18.318	-	-	-
monoanion I	-12.076 (C_4)	-15.450 (O_1)	+8.315	-	-	-
monoanion II	-11.443 (C_4)	-12.469 (N_2)	+4.116	-	-	-

Atomic labels according to Gaussian 09-see Figure 1. *SPL reaction corresponding to monocation+OH^-→neutral +H_2O; ** SPL reaction corresponding to neutral+OH^-→monoanion II+H_2O.

Table S2. M06-2X/6-311+G(d,p)/PCM(water) enthalpies [kcal/mol] of the reactions involved in the FHT, SET, SET-PT, SPL-ET, and SPL-FHT antioxidant mechanisms of harmalol towards HOO• radicals.

harmalol	RAF ΔH [kcal/mol]	FHT ΔH [kcal/mol]	SET ΔH [kcal/mol]	SET-PT ΔH [kcal/mol]	SPL-FHT ΔH [kcal/mol]	SPL-ET ΔH [kcal/mol]
monocation	-12.755 (C_4)	-3.005 (O_1)	+49.729	(1)+49.729<br>(2)-49.955 (O_1)	(1)*-35.673<br>(2)-9.439	(1)* -35.673<br>(2)+33.532
neutral	-9.039 (C_4)	-9.439 (O_1)	+33.532	(1)+33.532<br>(2)-39.297 (O_1)	(1)**-20.247<br>(2)-12.467	(1)**-20.247<br>(2)+4.118
zwitterion I	-21.850 (C_4)	-12.924 (O_1)	+23.634	-	-	-
zwitterion II	-11.007 (C_4)	-9.349 (N_2)	+18.316	-	-	-
monoanion I	-12.669 (C_4)	-15.449 (O_1)	+8.316	-	-	-
monoanion II	-12.036 (C_4)	-12.467 (N_2)	+4.118	-	-	-

Atomic labels according to Gaussian 09-see Figure 1. *SPL reaction corresponding to monocation+OH^-→neutral +H_2O; ** SPL reaction corresponding to neutral+OH^-→monoanion II+H_2O.

Table S3. M06-2X/6-311+G(d,p)/PCM(water) entropies [cal/mol K] (at 298.15 K) of the reactions involved in the FHT, SET, SET-PT, SPL-ET, and SPL-FHT antioxidant mechanisms of harmalol towards HOO• radicals.

harmalol	RAF ΔS [cal/mol K]	FHT ΔS [cal/mol K]	SET ΔS [cal/mol K]	SET-PT ΔS [cal/mol K]	SPL-FHT ΔS [cal/mol K]	SPL-ET ΔS [cal/mol K]
monocation	-42.702 (C_4)	-0.101 (O_1)	-0.790	(1) -0.790<br>(2)1.747 (O_1)	(1)*1.809<br>(2)-0.225	(1)*1.809<br>(2)-0.013
neutral	-41.610 (C_4)	-0.225(O_1)	-0.013	(1)-0.013<br>(2)0.793 (O_1)	(1)**2.757<br>(2) 0.469	(1)** 2.757<br>(2) +0.347
zwitterion I	-42.590 (C_4)	0.143 (O_1)	+1.052	-	-	-
zwitterion II	-43.304 (C_4)	-2.782 (N_2)	-0.644	-	-	-
monoanion I	-42.122 (C_4)	0.642 (O_1)	+0.800	-	-	-
monoanion II	-41.355 (C_4)	0.469 (N_2)	+0.347	-	-	-

Atomic labels according to Gaussian 09-see Figure 1. *SPL reaction corresponding to monocation+OH^-→neutral +H_2O; ** SPL reaction corresponding to neutral+OH^-→monoanion II+H_2O.

Table S4. Gibbs free energies (ΔG, kcal/mol) of RAF reactions of harmalol with HOO• radicals at different reaction sites, calculated at the M06-2X/6-311+G(d,p)/PCM(water) level at 298.15 K.

RAF	ΔG [kcal/mol]					
	**monocation**	**neutral**	**zwitterion I**	**zwitterion II**	**monoanion I**	**monoanion II**
**C4**	**-1.913**	**1.477**	**-11.042**	**+0.0145**	**-2.000**	**-1.596**
**C5**	16.085	8.579	+19.661	16.654	+10.322	4.462
**C11**	8.828	9.529	+3.751	10.217	+4.467	8.296
**C12**	11.026	10.348	+2.875	6.798	+4.208	0.538
**C14**	15.304	16.010	+8.543	1.040	+4.517	3.540

Atomic labels according to Gaussian 09.

Table S5 Key geometrical parameters of the stationary points: (a) reactant complex (RC), b) transition state (TS) and (c) product complex (PC)) encountered along the RAF reaction pathway of harmalol (monocation) with hydroperoxyl radicals. Atomic labels according to Gaussian 09.

monocation RAF RC		monocation RAF TS		monocation RAF PC	
**geometrical parameter**	**value**	**geometrical parameter**	**value**	**geometrical parameter**	**value**
C_4O_{30}	2.98	C_4O_{30}	2.00	C_4O_{30}	1.44 Å
$<O_{30}C_4C_5$	86.0	$<O_{30}C_4C_5$	97.0	$<O_{30}C_4C_5$	111.0°

Table S6 Key geometrical parameters of the stationary points: (a) reactant complex (RC), b) transition state (TS) and (c) product complex (PC)) encountered along the RAF reaction pathway of harmalol (neutral) with hydroperoxyl radicals. Atomic labels according to Gaussian 09.

neutral RAF RC		neutral RAF TS		neutral RAF PC	
**geometrical parameter**	**value**	**geometrical parameter**	**value**	**geometrical parameter**	**value**
C_4O_{29}	3.14	C_4O_{29}	1.98	C_4O_{29}	1.45 Å
$<O_{29}C_4C_5$	103.9	$<O_{29}C_4C_5$	103.0	$<O_{29}C_4C_5$	112.4°

Table S7 Key geometrical parameters of the stationary points: (a) reactant complex (RC), b) transition state (TS) and (c) product complex (PC)) encountered along the RAF reaction pathway of harmalol (zwitterion I) with hydroperoxyl radicals. Atomic labels according to Gaussian 09.

zwitterion I RAF RC		zwitterion I RAF TS		zwitterion I RAF PC	
**geometrical parameter**	**value**	**geometrical parameter**	**value**	**geometrical parameter**	**value**
C_4O_{29}	3.20 Å	C_4O_{29}	2.13 Å	C_4O_{29}	1.44 Å
$< O_{29}C_4C_5$	99.3 °	$<O_{29}C_4C_5$	98.4 °	$<O_{29}C_4C_5$	112.8°

Table S8 Key geometrical parameters of the stationary points: (a) reactant complex (RC), b) transition state (TS) and (c) product complex (PC)) encountered along the RAF reaction pathway of harmalol (monoanion I) with hydroperoxyl radicals. Atomic labels according to Gaussian 09.

monoanion I RAF RC		monoanion I RAF TS		monoanion I RAF PC	
**geometrical parameter**	**value**	**geometrical parameter**	**value**	**geometrical parameter**	**value**
C_4O_{28}	3.39	C_4O_{28}	2.02	C_4O_{28}	1.46 Å°
$<O_{28}C_4C_5$	101.1	$<O_{28}C_4C_5$	103.4	$<O_{28}C_4C_5$	113.6 °

Table S9 Key geometrical parameters of the stationary points: (a) reactant complex (RC), b) transition state (TS) and (c) product complex (PC)) encountered along the RAF reaction pathway of harmalol (monoanion II) with hydroperoxyl radicals. Atomic labels according to Gaussian 09.

monoanion II RAF RC		monoanion II RAF TS		monoanion II RAF PC	
**geometrical parameter**	**value**	**geometrical parameter**	**value**	**geometrical parameter**	**value**
C_4O_{28}	3.21	C_4O_{28}	2.05	C_4O_{28}	1.46 Å
$<O_{28}C_4C_5$	104.5	$<O_{28}C_4C_5$	97.4	$<O_{28}C_4C_5$	112.0 °

Table S10 Evolution of UM06-2X/6-311+G(d,p)/PCM(water) calculated natural charges along the intrinsic reaction coordinate for the formal hydrogen transfer (FHT) antioxidant mechanism of harmalol (monocationic form).

	REV40	REV10	REV4	REV3	REV2	TS	FOR2	FOR4	FOR6	FOR26
**O1**	**-0.34786**	**-0.34694**	**-0.34315**	**-0.34677**	**-0.33677**	**-0.24756**	**-0.18477**	**-0.17855**	**-0.17724**	**-0.17716**
N2	-0.26251	-0.26267	-0.26191	-0.26176	-0.25876	-0.22579	-0.19861	-0.19889	-0.20007	-0.19796
N3	-0.24387	-0.24387	-0.24292	-0.24374	-0.23880	-0.19466	-0.15804	-0.15581	-0.15667	-0.15767
C4	0.02667	0.02665	0.02403	0.02702	0.02178	0.00712	-0.01471	-0.02563	-0.02864	-0.02890
C5	0.02277	0.02297	0.02525	0.02197	0.03007	0.09808	0.16271	0.16054	0.15710	0.15443
C6	-0.20324	-0.20337	-0.20307	-0.20324	-0.20297	-0.20994	-0.20832	-0.20748	-0.20741	-0.20730
C7	-0.06850	-0.06849	-0.06692	-0.06775	-0.06193	-0.01772	0.02925	0.03879	0.04100	0.03989
C8	0.11405	0.11392	0.11227	0.11457	0.10901	0.08199	0.05907	0.05041	0.04786	0.04429
C9	-0.08940	-0.08932	-0.08917	-0.08946	-0.08941	-0.08984	-0.09170	-0.09192	-0.09183	-0.09165
C10	0.18641	0.18650	0.18561	0.18534	0.18590	0.19174	0.19565	0.19666	0.19744	0.19789
C11	-0.07441	-0.07466	-0.07621	-0.07700	-0.07870	-0.07472	-0.07372	-0.08867	-0.09454	-0.09439
C12	-0.16808	-0.16480	-0.16321	-0.16366	-0.15043	-0.04364	0.04109	0.05688	0.06178	0.06914
C13	-0.31396	-0.31368	-0.31402	-0.31361	-0.31378	-0.31457	-0.31532	-0.31476	-0.31475	-0.31506
C14	-0.13458	-0.13071	-0.12891	-0.12881	-0.12674	-0.11105	-0.09672	-0.08461	-0.08037	-0.08214
C15	0.18511	0.17742	0.17924	0.17603	0.18148	0.21188	0.24225	0.22590	0.21787	0.21411
H16	0.12139	0.12130	0.12101	0.12122	0.12116	0.12423	0.12617	0.12538	0.12503	0.12489
H17	0.11548	0.11550	0.11517	0.11536	0.11520	0.11899	0.12193	0.12031	0.11974	0.11959
H18	0.21800	0.21797	0.21773	0.21792	0.21806	0.22185	0.22437	0.22376	0.22353	0.22322
H19	0.10729	0.10732	0.10734	0.10722	0.10770	0.11335	0.11797	0.11730	0.11701	0.11690
H20	0.11549	0.11547	0.11521	0.11513	0.11530	0.11813	0.12036	0.11973	0.11937	0.11930
H21	0.11341	0.11342	0.11270	0.11342	0.11354	0.11638	0.11832	0.11798	0.11803	0.11771
H22	0.11695	0.11823	0.11868	0.11860	0.11806	0.12167	0.12441	0.12231	0.12076	0.11871
H23	0.12492	0.12530	0.12516	0.12499	0.12514	0.12748	0.12937	0.12877	0.12862	0.12857
H24	0.11780	0.11744	0.11759	0.11783	0.11792	0.11975	0.12120	0.12090	0.12075	0.12068
H25	0.12662	0.12629	0.12616	0.12600	0.12615	0.12871	0.13074	0.13015	0.13003	0.13017
H26	0.11408	0.11406	0.11319	0.11394	0.11345	0.11549	0.11658	0.11520	0.11437	0.11402
**H27**	**0.24757**	**0.24738**	**0.23959**	**0.23536**	**0.23370**	**0.23462**	**0.23208**	**0.23326**	**0.23720**	**0.24365**
H28	0.21484	0.21478	0.21460	0.21443	0.21437	0.21674	0.21863	0.21755	0.21721	0.21704
O29	0.01254	0.00940	0.01035	0.03567	0.01553	-0.12829	-0.25582	-0.25448	-0.24903	-0.24520
**O30**	**0.26136**	**0.26342**	**0.26078**	**0.24827**	**0.22615**	**-0.05184**	**-0.27082**	**-0.25991**	**-0.25356**	**-0.25350**
H31	0.24369	0.24378	0.24785	0.24553	0.24864	0.24145	0.23639	0.23892	0.23939	0.23674

REV and FOR denote points along the intrinsic reaction coordinate (IRC) before and after the transition state (TS), respectively. REV40, REV2, FOR2, FOR 26 correspond to reactant complex (RC), pre-transition state (pre-TS), post-transition state (post-TS), and product complex (PC) (Figure 12 and Table 8), respectively.

Table S11 Evolution of UM062X/6-311+G(d,p)/PCM(water) calculated spin density along the intrinsic reaction coordinate for the formal hydrogen transfer (FHT) antioxidant mechanism of harmalol (monocationic form).

	REV40	REV10	REV4	REV3	REV2	TS	FOR2	FOR4	FOR6	FOR26
**O1**	**0.000083**	**0.000194**	**0.008971**	**0.007127**	**0.027758**	**0.130418**	**0.194152**	**0.218417**	**0.228897**	**0.232945**
N2	0.000083	0.000110	0.001558	0.000789	0.005268	0.043563	0.076806	0.078186	0.077186	0.079184
N3	0.000178	0.000195	0.002197	0.001145	0.007199	0.055687	0.096633	0.103929	0.104369	0.103356
C4	-0.000124	-0.000136	-0.001199	-0.000758	-0.003455	-0.014574	-0.020227	-0.031261	-0.034061	-0.034582
C5	0.000194	0.000210	0.002595	0.001187	0.008676	0.076628	0.140317	0.140766	0.137904	0.135582
C6	-0.000005	-0.000005	-0.000024	-0.000025	-0.000036	0.000879	0.001979	0.001120	0.000767	0.000668
C7	0.000174	0.000184	0.001947	0.001063	0.006181	0.043729	0.077786	0.085344	0.086900	0.086259
C8	-0.000074	-0.000085	-0.000559	-0.000395	-0.001542	-0.006410	-0.009622	-0.015041	-0.016730	-0.018161
C9	0.000006	0.000007	0.000093	0.000044	0.000319	0.002862	0.005082	0.005095	0.004962	0.004899
C10	0.000094	0.000102	0.000980	0.000580	0.002982	0.018418	0.031827	0.038907	0.040651	0.041160
C11	-0.000170	-0.000170	-0.000835	-0.000689	-0.001860	0.000785	0.004219	-0.007084	-0.010780	-0.010266
C12	0.000268	0.000343	0.005430	0.003090	0.017734	0.122288	0.210546	0.229514	0.234077	0.240400
C13	0.000007	0.000008	0.000072	0.000041	0.000214	0.001323	0.002321	0.002857	0.002991	0.002943
C14	0.000120	0.000124	0.001354	0.000905	0.004071	0.021251	0.037926	0.048711	0.052719	0.052819
C15	-0.000239	-0.000221	0.000384	-0.000357	0.002538	0.041903	0.075874	0.061939	0.056167	0.052700
H16	-0.000004	-0.000004	-0.000041	-0.000024	-0.000120	-0.000639	-0.001000	-0.001280	-0.001357	-0.001305
H17	-0.000009	-0.000010	-0.000079	-0.000054	-0.000212	-0.000439	-0.000300	-0.001380	-0.001748	-0.001718
H18	-0.000001	0.000001	0.000063	0.000032	0.000214	0.001801	0.003244	0.003287	0.003225	0.003324
H19	0.000010	0.000010	0.000126	0.000063	0.000422	0.003511	0.006140	0.006343	0.006250	0.006229
H20	0.000001	0.000001	0.000014	0.000007	0.000046	0.000364	0.000632	0.000701	0.000699	0.000738
H21	-0.000016	-0.000016	-0.000072	-0.000056	-0.000169	-0.000263	-0.000207	-0.000915	-0.001124	-0.001061
H22	-0.000182	-0.000128	0.000127	0.000226	0.000547	0.005100	0.010640	0.012060	0.012472	0.013022
H23	0.000003	0.000003	0.000023	0.000015	0.000061	0.000252	0.000452	0.000714	0.000797	0.000804
H24	0.000000	0.000000	0.000003	0.000002	0.000006	0.000018	0.000020	0.000048	0.000057	0.000049
H25	0.000004	0.000004	0.000037	0.000024	0.000107	0.000540	0.000920	0.001289	0.001396	0.001424
H26	-0.000004	-0.000004	0.000026	0.000017	0.000115	0.000534	0.001096	0.001746	0.001988	0.002007
**H27**	**0.002461**	**0.002088**	**0.004235**	**0.005528**	**0.005410**	**0.000753**	**0.002392**	**0.002442**	**0.001930**	**0.001568**
H28	0.000009	0.000010	0.000110	0.000059	0.000360	0.002693	0.004620	0.005069	0.005123	0.005060
O29	0.317081	0.314866	0.310949	0.334300	0.309913	0.151231	0.014881	0.002870	0.001597	-0.000144
**O30**	**0.671736**	**0.673980**	**0.653383**	**0.637136**	**0.599338**	**0.292849**	**0.030800**	**0.005662**	**0.002727**	**0.000058**
H31	0.008315	0.008339	0.008134	0.008981	0.007914	0.002941	0.000045	-0.000067	-0.000060	0.000037

REV and FOR denote points along the intrinsic reaction coordinate (IRC) before and after the transition state (TS), respectively. REV40, REV2, FOR2, FOR 26 correspond to reactant complex (RC), pre-transition state (pre-TS), post-transition state (post-TS), and product complex (PC) (Figure 12 and Table 8), respectively.

Table S12 Evolution of UM06-2X/6-311+G(d,p)/PCM(water) calculated natural charges along the intrinsic reaction coordinate for the formal hydrogen transfer (FHT) antioxidant mechanism of harmalol (neutral form).

	REV14	REV7	REV5	REV2	TS	FOR2	FOR3	FOR4	FOR5	FOR39
**O1**	**-0.35749**	**-0.35655**	**-0.35597**	**-0.35309**	**-0.28754**	**-0.23718**	**-0.22975**	**-0.21755**	**-0.21713**	**-0.21516**
N2	-0.26871	-0.26869	-0.26842	-0.26782	-0.24794	-0.23225	-0.23196	-0.23635	-0.23537	-0.23682
N3	-0.25501	-0.25502	-0.25415	-0.25333	-0.19304	-0.13360	-0.13454	-0.13959	-0.14313	-0.14831
C4	-0.02146	-0.02147	-0.02148	-0.01234	0.00306	0.03722	0.02542	0.00885	-0.00310	-0.01428
C5	0.04032	0.04057	0.04226	0.04153	0.13118	0.21964	0.22174	0.21256	0.21167	0.20344
C6	-0.20658	-0.20674	-0.20672	-0.20858	-0.20921	-0.21215	-0.21175	-0.21050	-0.20974	-0.20893
C7	-0.06136	-0.06134	-0.06065	-0.06417	-0.02846	-0.01334	-0.00571	0.00727	0.01468	0.02215
C8	0.09331	0.09278	0.09251	0.09448	0.09530	0.10396	0.09676	0.08919	0.08062	0.07325
C9	-0.10082	-0.10069	-0.10071	-0.10133	-0.10457	-0.10768	-0.10748	-0.10758	-0.10731	-0.10710
C10	0.12613	0.12603	0.12558	0.12829	0.10636	0.08413	0.08291	0.08461	0.08512	0.08685
C11	-0.07897	-0.07912	-0.07938	-0.08690	-0.06805	-0.03499	-0.04498	-0.05785	-0.06761	-0.07838
C12	-0.15931	-0.15945	-0.15873	-0.15583	-0.10274	-0.06106	-0.05132	-0.03888	-0.02865	-0.01552
C13	-0.30881	-0.30867	-0.30873	-0.30913	-0.30906	-0.30885	-0.30858	-0.30829	-0.30831	-0.30849
C14	-0.14436	-0.14383	-0.14314	-0.13873	-0.12973	-0.12930	-0.12131	-0.11035	-0.10312	-0.09552
C15	0.15849	0.15818	0.15989	0.15960	0.21220	0.26967	0.26550	0.25133	0.24469	0.22912
H16	0.11154	0.11142	0.11134	0.11150	0.11564	0.11996	0.11920	0.11849	0.11754	0.11672
H17	0.10407	0.10403	0.10409	0.10400	0.11244	0.12179	0.12005	0.11778	0.11597	0.11430
H18	0.21258	0.21252	0.21253	0.21286	0.21674	0.22080	0.22033	0.21988	0.21905	0.21838
H19	0.09653	0.09662	0.09671	0.09701	0.10450	0.11218	0.11157	0.11049	0.10952	0.10858
H20	0.09859	0.09858	0.09862	0.09865	0.10212	0.10559	0.10524	0.10467	0.10406	0.10361
H21	0.10836	0.10824	0.10816	0.10914	0.11222	0.11411	0.11370	0.11395	0.11309	0.11291
H22	0.11195	0.11388	0.11429	0.11406	0.12060	0.12624	0.12456	0.12290	0.12036	0.11503
H23	0.11026	0.11020	0.11020	0.11012	0.11136	0.11259	0.11228	0.11197	0.11167	0.11134
H24	0.11175	0.11169	0.11170	0.11172	0.11368	0.11559	0.11535	0.11509	0.11471	0.11449
H25	0.10997	0.10992	0.10994	0.10995	0.11112	0.11226	0.11195	0.11168	0.11140	0.11153
H26	0.11018	0.11007	0.10973	0.10997	0.11291	0.11533	0.11400	0.11323	0.11138	0.11006
**H27**	**0.24577**	**0.24485**	**0.24375**	**0.23763**	**0.24312**	**0.24191**	**0.23518**	**0.23201**	**0.23242**	**0.24451**
O28	0.00989	0.00570	0.00379	0.01800	-0.13391	-0.27567	-0.27640	-0.26367	-0.26010	-0.24622
**O29**	**0.26034**	**0.26287**	**0.25805**	**0.23785**	**-0.04813**	**-0.31864**	**-0.30597**	**-0.29190**	**-0.27221**	**-0.25735**
H30	0.24284	0.24343	0.24495	0.24488	0.23784	0.23175	0.23404	0.23656	0.23785	0.23584

REV and FOR denote points along the intrinsic reaction coordinate (IRC) before and after the transition state (TS), respectively. REV14, REV2, FOR2, FOR 39 correspond to reactant complex (RC), pre-transition state (pre-TS), post-transition state (post-TS), and product complex (PC) (Figure 13 and Table 8), respectively.

Table S13 Evolution of UM06-2X/6-311+G(d,p)/PCM(water) calculated spin density along the intrinsic reaction coordinate for the formal hydrogen transfer (FHT) antioxidant mechanism of harmalol (neutral form).

	REV14	REV7	REV5	REV2	TS	FOR2	FOR3	FOR4	FOR5	FOR39
**O1**	**0.000932**	**0.001492**	**0.005056**	**0.012325**	**0.091485**	**0.145264**	**0.160289**	**0.188095**	**0.190839**	**0.218258**
N2	0.000110	0.000214	0.000772	0.001465	0.026173	0.050930	0.052550	0.047818	0.050068	0.047889
N3	0.000427	0.000625	0.002042	0.003975	0.066004	0.128286	0.130777	0.126217	0.126446	0.122176
C4	-0.000053	-0.000003	0.000291	0.000458	0.030316	0.068736	0.057541	0.042130	0.031793	0.021103
C5	0.000585	0.000853	0.002779	0.005380	0.089147	0.176012	0.180110	0.172534	0.174292	0.167278
C6	0.000010	0.000021	0.000097	0.000173	0.005146	0.010922	0.009847	0.008078	0.007041	0.005815
C7	0.000391	0.000523	0.001493	0.003130	0.032876	0.057609	0.064626	0.074319	0.081471	0.087079
C8	0.000004	-0.000016	0.000120	0.000219	0.014619	0.032716	0.028450	0.022291	0.017375	0.011144
C9	0.000023	0.000035	0.000125	0.000239	0.004821	0.009788	0.009594	0.008725	0.008362	0.007677
C10	-0.000053	-0.000080	-0.000272	-0.000528	-0.009635	-0.019052	-0.018671	-0.017983	-0.016908	-0.016104
C11	-0.000160	-0.000098	0.000076	0.000042	0.019309	0.041965	0.034292	0.024629	0.017000	0.009200
C12	0.000498	0.000820	0.002715	0.005722	0.062237	0.110887	0.124470	0.136337	0.152052	0.165126
C13	-0.000004	-0.000007	-0.000024	-0.000047	-0.000889	-0.001772	-0.001736	-0.001645	-0.001556	-0.001484
C14	0.000269	0.000323	0.000834	0.001943	0.011741	0.018609	0.027334	0.038044	0.046664	0.055617
C15	-0.000046	0.000160	0.001210	0.002362	0.055525	0.108571	0.102693	0.095028	0.085299	0.074564
H16	-0.000004	-0.000003	-0.000002	-0.000006	0.000544	0.001300	0.000972	0.000583	0.000302	0.000076
H17	0.000001	0.000009	0.000057	0.000098	0.003963	0.008726	0.007628	0.005929	0.004841	0.003677
H18	-0.000001	0.000006	0.000029	0.000054	0.001052	0.002151	0.002237	0.001920	0.002045	0.001902
H19	0.000027	0.000041	0.000138	0.000265	0.004944	0.009915	0.009890	0.009172	0.008938	0.008395
H20	0.000002	0.000004	0.000012	0.000025	0.000452	0.000940	0.000993	0.000961	0.000973	0.000949
H21	-0.000018	-0.000016	-0.000016	-0.000037	0.000841	0.002035	0.001569	0.000910	0.000449	-0.000046
H22	-0.000183	-0.000099	-0.000028	0.000131	0.002103	0.005313	0.006356	0.006936	0.008137	0.008954
H23	-0.000006	-0.000009	-0.000031	-0.000060	-0.001019	-0.001986	-0.001995	-0.001915	-0.001884	-0.001786
H24	0.000000	0.000000	-0.000002	-0.000004	-0.000079	-0.000147	-0.000151	-0.000141	-0.000144	-0.000151
H25	-0.000007	-0.000010	-0.000034	-0.000067	-0.001150	-0.002272	-0.002294	-0.002206	-0.002169	-0.002135
H26	0.000000	-0.000002	0.000003	0.000030	-0.000042	-0.000115	0.000396	0.000972	0.001549	0.002067
**H27**	**0.002127**	**0.001929**	**0.002459**	**0.004048**	**-0.000014**	**0.002535**	**0.003069**	**0.003050**	**0.002672**	**0.001982**
O28	0.314756	0.311073	0.307951	0.317449	0.153891	0.009320	0.002538	0.002636	0.001220	0.000119
**O29**	**0.672092**	**0.673880**	**0.664132**	**0.632919**	**0.332712**	**0.022849**	**0.006706**	**0.006661**	**0.002902**	**0.000617**
H30	0.008277	0.008332	0.008014	0.008293	0.002925	-0.000040	-0.000092	-0.000097	-0.000085	0.000049

REV and FOR denote points along the intrinsic reaction coordinate (IRC) before and after the transition state (TS), respectively. REV14, REV2, FOR2, FOR 39 correspond to reactant complex (RC), pre-transition state (pre-TS), post-transition state (post-TS), and product complex (PC) (Figure 13 and Table 8), respectively.

Cartesian coordinates of the structures

neutral form

**O**	**-4.62320700**	**-0.56687700**	**-0.05658200**
**N**	**0.20520000**	**-1.37950600**	**-0.12537600**
**N**	**3.54313200**	**0.17825500**	**-0.07212400**
**C**	**0.75632900**	**0.79906100**	**0.05131300**
**C**	**1.24963500**	**-0.47191100**	**-0.06431000**
**C**	**1.72137300**	**1.92653600**	**0.23946300**
**C**	**-0.67073600**	**0.69267700**	**0.06141900**
**C**	**-0.97568100**	**-0.68741500**	**-0.06717900**
**C**	**3.04938000**	**1.52079900**	**-0.41017500**
**C**	**2.68023100**	**-0.76100400**	**0.03373000**
**C**	**-1.72796900**	**1.61400100**	**0.15460200**
**C**	**-2.29257000**	**-1.16195300**	**-0.11961700**
**C**	**3.11362800**	**-2.17522300**	**0.29024700**

C	-3.02770700	1.15683300	0.11366500
C	-3.30237100	-0.22214200	-0.02430800
H	1.36609600	2.85207600	-0.21893600
H	1.85762500	2.12869900	1.30931200
H	0.29156800	-2.37620300	-0.25257000
H	2.92609100	1.54727400	-1.50148000
H	3.83106700	2.23758700	-0.15717700
H	-1.52570300	2.67432800	0.25515600
H	-2.51214300	-2.21849000	-0.22545300
H	2.65073100	-2.56259400	1.20148700
H	4.19689900	-2.21915500	0.38603600
H	2.80488500	-2.82126900	-0.53706700
H	-3.86540400	1.83997400	0.18188900
H	-4.71562700	-1.52000400	-0.15913700

neutral radical

O	-4.57428300	-0.68814700	-0.08580300
N	0.19940400	-1.40328900	-0.09829500
N	3.46703700	0.23507300	-0.10999300
C	0.66877500	0.79819900	0.08604700
C	1.20251700	-0.48597300	-0.02056900
C	1.61664000	1.94020000	0.25765500
C	-0.72273500	0.66436700	0.07019500
C	-1.01241500	-0.73663900	-0.05783700
C	2.94062500	1.56995000	-0.42147000
C	2.64344000	-0.73381100	0.03148400
C	-1.80631100	1.59393800	0.14688200
C	-2.28876400	-1.23164700	-0.11731700
C	3.12783500	-2.13452100	0.26327500
C	-3.08004100	1.13294800	0.09329300
C	-3.39186100	-0.29556000	-0.04084000
H	1.22760600	2.86337900	-0.17513700
H	1.77814300	2.12362700	1.32623800
H	0.31120600	-2.40161800	-0.20178500
H	2.80949900	1.60852100	-1.51045100
H	3.71118400	2.29706500	-0.16634800
H	-1.59832500	2.65343800	0.24461900

H	-2.50676000	-2.28828100	-0.21365600
H	2.70107300	-2.54536700	1.18154800
H	4.21362000	-2.14010200	0.33323600
H	2.82380200	-2.78205900	-0.56435800
H	-3.92811200	1.80538300	0.14574100

monocation

O	4.63996100	-0.54514900	0.03700800
N	-0.16076400	-1.40458200	0.12908100
N	-3.42241600	0.21156600	0.02577200
C	-0.71492200	0.78689900	-0.02479500
C	-1.21034600	-0.50130600	0.09001300
C	-1.65619000	1.93137900	-0.22493900
C	0.69554400	0.67972300	-0.04658100
C	1.00636400	-0.70339600	0.07237000
C	-3.00068800	1.57215800	0.40070500
C	-2.59199300	-0.79669700	-0.04170300
C	1.74829200	1.61610100	-0.14624600
C	2.32673100	-1.17163900	0.11505200
C	-3.09180500	-2.17888600	-0.27164600
C	3.04077600	1.16466100	-0.11523400
C	3.32412400	-0.22274500	0.01647300
H	-1.28786300	2.84496700	0.24327100
H	-1.76186300	2.13174400	-1.29673100
H	-0.23675500	-2.39948300	0.28066900
H	-2.92247800	1.59238200	1.49210000
H	-3.78604000	2.25651600	0.09034400
H	1.53613300	2.67428400	-0.24100800
H	2.55311700	-2.22668700	0.21341500
H	-2.60210500	-2.60700600	-1.14795800
H	-4.17082700	-2.19595300	-0.41062000
H	-2.83560700	-2.79676400	0.59297200
H	3.87845100	1.84697600	-0.18536200
H	4.75607000	-1.49710100	0.13333700
H	-4.41296900	0.03091700	-0.08527400

monocation radical

O	-4.59682900	-0.67478300	-0.07312100
N	0.15585800	-1.42627300	-0.09737600

N	3.35431600	0.26038400	-0.06532600
C	0.63247400	0.78533800	0.06359900
C	1.16346800	-0.51017000	-0.03858400
C	1.55849500	1.94432700	0.24912000
C	-0.75243600	0.65053200	0.05839100
C	-1.04231900	-0.75031000	-0.05808100
C	2.89383100	1.61880000	-0.41352300
C	2.56549700	-0.77262700	0.03881300
C	-1.83039300	1.59527500	0.13864500
C	-2.32448600	-1.23983300	-0.11030900
C	3.10767400	-2.14124100	0.24264600
C	-3.10270300	1.14534100	0.09325000
C	-3.42140100	-0.28724900	-0.03386800
H	1.15970000	2.85613600	-0.19547100
H	1.69502600	2.12933800	1.31935000
H	0.25817400	-2.42563100	-0.20981900
H	2.79927400	1.64983800	-1.50256500
H	3.67305700	2.31192400	-0.10763800
H	-1.61005700	2.65231000	0.22996800
H	-2.55371000	-2.29429300	-0.19863900
H	2.65345300	-2.59019100	1.12759300
H	4.19002900	-2.12875800	0.35329900
H	2.84595600	-2.75734100	-0.62200100
H	-3.94857500	1.81973100	0.14572800
H	4.35488600	0.10732300	0.00349500

zwitterion I

O	4.63073900	-0.57534600	0.03940500
N	-0.16207100	-1.46503300	0.13952600
N	-3.41155200	0.16762400	0.02813400
C	-0.71697700	0.79710100	-0.02686500
C	-1.17318300	-0.53272100	0.07990000
C	-1.68448700	1.92012800	-0.23932700
C	0.67775600	0.69292000	-0.04149600
C	0.95370500	-0.72362400	0.07992200
C	-3.01559500	1.53321600	0.39980300
C	-2.54889300	-0.82975100	-0.02851200
C	1.74830700	1.61849500	-0.14133100
C	2.30327500	-1.17611700	0.11825100
C	-3.04399200	-2.21674400	-0.25680600
C	3.03488400	1.15444100	-0.11108900
C	3.30217200	-0.24433100	0.02014900
H	-1.34159600	2.85404500	0.21027400
H	-1.81810200	2.10594900	-1.31204600
H	-2.91895400	1.56002000	1.49083700
H	-3.81947500	2.20334200	0.10253000
H	1.55287300	2.68135900	-0.23682500

H	2.52830400	-2.23345700	0.21697100
H	-2.86583300	-2.49065100	-1.29949400
H	-4.10758400	-2.30828800	-0.04011300
H	-2.47204600	-2.90610000	0.36337400
H	3.88120800	1.82728000	-0.18078700
H	4.72724500	-1.52901200	0.13314300
H	-4.39840300	-0.03794200	-0.05230900

zwitterion I radical

O	-4.58227700	-0.71404300	-0.07442700
N	0.16528900	-1.48036800	-0.10495900
N	3.34605800	0.21451200	-0.06575900
C	0.63163400	0.79926200	0.06658800
C	1.12925500	-0.54339200	-0.03521600
C	1.58710100	1.93302500	0.26048700
C	-0.73762000	0.67132500	0.05622300
C	-0.99641100	-0.75527400	-0.06274600
C	2.91057200	1.57712200	-0.41397000
C	2.52460400	-0.80712900	0.02763800
C	-1.83551000	1.59460900	0.13390500
C	-2.29033900	-1.23909300	-0.11193600
C	3.05781200	-2.18141000	0.23177000
C	-3.10217300	1.12321600	0.08784600
C	-3.40170300	-0.31475800	-0.03699700
H	1.21388400	2.86677000	-0.16263900
H	1.74765400	2.09894200	1.33176100
H	2.79793100	1.61353100	-1.50194200
H	3.70631500	2.25865200	-0.12225800
H	-1.64129600	2.65834200	0.22481000
H	-2.50370900	-2.29856500	-0.19899300
H	2.83196000	-2.50403500	1.25060100
H	4.13316900	-2.22711100	0.06693000
H	2.54526600	-2.86434900	-0.44626600
H	-3.95748200	1.78707900	0.13851200
H	4.34175400	0.03905200	-0.01161700

zwitterion II

O	-4.64403200	-0.64041600	-0.05143400
N	0.12984900	-1.43443300	-0.09249100
N	3.37723400	0.27008000	0.01580500
C	0.64632000	0.77743500	0.02060100
C	1.18611800	-0.51827600	-0.07893600
C	1.57137800	1.93680200	0.21877000
C	-0.73818800	0.65555200	0.04509900
C	-1.05397400	-0.74553100	-0.06102400
C	2.92191300	1.59553900	-0.40766000
C	2.54528500	-0.77189600	0.05448300
C	-1.81093200	1.59106100	0.13795600
C	-2.35270700	-1.22053000	-0.10877100
C	3.10776700	-2.14388300	0.22959000
C	-3.08909200	1.13438400	0.10338600
C	-3.43421300	-0.29165700	-0.02448000
H	1.18334300	2.84847500	-0.23745600
H	1.68136700	2.12720700	1.29269100
H	0.21004900	-2.41338500	-0.32018900

H	2.82095200	1.58655600	-1.49956800
H	3.68474800	2.31997500	-0.13106600
H	-1.59826900	2.65131800	0.22725000
H	-2.56951900	-2.27895400	-0.19208500
H	2.45623200	-2.75065200	0.85802800
H	4.10004100	-2.10268000	0.67806400
H	3.19146600	-2.62767900	-0.74749000
H	-3.92590200	1.82185900	0.16608300
H	4.37011500	0.09776600	0.07993200

zwitterion II radical

O	4.58227000	-0.71403900	0.07440200
N	-0.16530500	-1.48036400	0.10534600
N	-3.34602100	0.21453200	0.06571300
C	-0.63165300	0.79920300	-0.06648800
C	-1.12924500	-0.54341000	0.03548100
C	-1.58701800	1.93306300	-0.26027300
C	0.73762400	0.67128000	-0.05619700
C	0.99642100	-0.75527500	0.06299300
C	-2.91052300	1.57707200	0.41410300
C	-2.52462800	-0.80712200	-0.02772300
C	1.83547600	1.59456200	-0.13420400
C	2.29035200	-1.23906400	0.11218200
C	-3.05789900	-2.18132400	-0.23222700
C	3.10215500	1.12317600	-0.08819100
C	3.40168100	-0.31475400	0.03688400
H	-1.21376100	2.86667300	0.16312300
H	-1.74750000	2.09926800	-1.33150700
H	-2.79782100	1.61329900	1.50207300
H	-3.70628100	2.25865500	0.12255900
H	1.64124800	2.65827500	-0.22528500
H	2.50374900	-2.29851000	0.19946800
H	-2.83211100	-2.50379100	-1.25113000
H	-4.13323300	-2.22701900	-0.06732600
H	-2.54537700	-2.86441900	0.44567500
H	3.95744700	1.78704000	-0.13909700
H	-4.34170300	0.03922800	0.01078100

monoanion I

O	-4.61299700	-0.60148300	-0.06423200
N	0.21073800	-1.44250000	-0.13552800
N	3.53182700	0.13399700	-0.08302000
C	0.75783900	0.80360200	0.05746300
C	1.21051200	-0.50623100	-0.05792300
C	1.74683200	1.91106800	0.25052900
C	-0.65618700	0.70406700	0.06318400
C	-0.92568000	-0.70746100	-0.07397600
C	3.05587400	1.48261800	-0.42085500
C	2.64374400	-0.78969000	0.02825900
C	-1.72776700	1.61319600	0.15882700
C	-2.26588800	-1.16414200	-0.12580900
C	3.07964300	-2.20499800	0.28700600
C	-3.02626700	1.14574500	0.11502700
C	-3.28228800	-0.23966900	-0.02874700
H	1.41255600	2.85564800	-0.18843700
H	1.91699600	2.10206000	1.31988500
H	2.90753700	1.50972700	-1.51025800
H	3.85634700	2.18799400	-0.18884500
H	-1.54181600	2.67791300	0.26421300

H	-2.48551300	-2.22277100	-0.23514300
H	2.67156600	-2.56363000	1.23531200
H	4.16696300	-2.26619600	0.30991500
H	2.69072200	-2.86352900	-0.49330200
H	-3.87122900	1.82096600	0.18451700
H	-4.68030300	-1.55543400	-0.17207200

monoanion I radical

O	-4.56346000	-0.73242800	-0.09650700
N	0.21583600	-1.45906700	-0.10831300
N	3.45830500	0.18997200	-0.12414300
C	0.66843200	0.80695800	0.09733000
C	1.16579300	-0.52497100	-0.01822200
C	1.64344200	1.92429600	0.27029400
C	-0.71181400	0.68238200	0.07654000
C	-0.96725000	-0.73958700	-0.06156500
C	2.94541000	1.53004400	-0.43832500
C	2.61364600	-0.76425000	0.02484600
C	-1.81304300	1.58968100	0.15382800
C	-2.25509600	-1.22748900	-0.12397500
C	3.09694800	-2.16509300	0.26673400
C	-3.08393400	1.10814800	0.09434400
C	-3.37444400	-0.32121000	-0.04668900
H	1.27263300	2.86921300	-0.13419700
H	1.84212700	2.08729900	1.33773000
H	2.78244900	1.56691900	-1.52363200
H	3.73367700	2.24893300	-0.21019600
H	-1.63090300	2.65519300	0.25731900
H	-2.45717700	-2.28884000	-0.22604500
H	2.72867400	-2.53458800	1.22689400
H	4.18531300	-2.19324200	0.25609700
H	2.70345700	-2.83306800	-0.50302900
H	-3.94009400	1.77180700	0.14745900

monoanion II

O	-4.64140100	-0.66412200	-0.05251600
N	0.17294800	-1.38882300	-0.13491700
N	3.50943900	0.20133600	-0.04051100
C	0.70734700	0.79563100	0.02481300
C	1.22045100	-0.47120400	-0.09895400
C	1.66349800	1.92758000	0.23446100
C	-0.71435700	0.67912600	0.05281200
C	-1.02061700	-0.70414200	-0.07466200
C	3.00461100	1.53380700	-0.39606600
C	2.64069600	-0.74339700	0.03628700
C	-1.78946000	1.58893600	0.15294300
C	-2.32754100	-1.19318100	-0.12398400
C	3.08194400	-2.15582000	0.29937600
C	-3.08010100	1.12073300	0.11839000
C	-3.41735800	-0.28804000	-0.02362400
H	1.31146100	2.85580600	-0.22210100
H	1.78079900	2.12368100	1.30865000
H	0.26185100	-2.37606600	-0.31616000
H	2.88480000	1.55181000	-1.48908500
H	3.77398600	2.26490400	-0.14374300
H	-1.59414800	2.65276500	0.25300300
H	-2.52711400	-2.25457000	-0.22842800
H	2.59884900	-2.55207500	1.19619300
H	4.16292200	-2.19130000	0.42309000

H	2.80015800	-2.80205300	-0.53744800
H	-3.91375300	1.81231100	0.19340600

monoanion II radical

O	-4.56345200	-0.73240700	-0.09618000
N	0.21580500	-1.45895700	-0.10845900
N	3.45835200	0.18992800	-0.12395200
C	0.66848300	0.80692200	0.09724100
C	1.16579900	-0.52488500	-0.01847400
C	1.64347100	1.92426100	0.27044000
C	-0.71179500	0.68239900	0.07643200
C	-0.96725500	-0.73951100	-0.06159300
C	2.94544900	1.52999700	-0.43816400
C	2.61358800	-0.76427500	0.02474900
C	-1.81301600	1.58967400	0.15362300
C	-2.25506400	-1.22753100	-0.12389600
C	3.09680800	-2.16513000	0.26674100
C	-3.08391100	1.10815800	0.09423800
C	-3.37439700	-0.32120700	-0.04677600
H	1.27272100	2.86919700	-0.13405500
H	1.84199200	2.08718200	1.33791900
H	2.78236500	1.56682900	-1.52346000
H	3.73371400	2.24890200	-0.21011500
H	-1.63094800	2.65520500	0.25708800
H	-2.45714700	-2.28889300	-0.22582500
H	2.72878400	-2.53446200	1.22706300
H	4.18516500	-2.19338200	0.25577900
H	2.70299500	-2.83317000	-0.50280300
H	-3.94006800	1.77181700	0.14736900

monocation RAF RC

O	4.67438800	-0.62218100	-0.09868000
N	-0.11947000	-1.49532800	0.12924900
N	-3.39110400	0.00574800	-0.41138600
C	-0.68981600	0.55543000	-0.64560800
C	-1.17420000	-0.65217200	-0.17664400
C	-1.63634100	1.60190400	-1.13697900
C	0.72277900	0.46399300	-0.61956600
C	1.04342400	-0.82618300	-0.11043100
C	-2.97487800	1.42030600	-0.42743800
C	-2.55737200	-0.97940700	-0.20971500
C	1.76991200	1.35498300	-0.94709100
C	2.36657400	-1.24185800	0.09245200
C	-3.04781200	-2.37504300	-0.05647700
C	3.06560100	0.95168900	-0.75771600
C	3.35808100	-0.34083000	-0.24190300
H	-1.26648400	2.60604100	-0.92573200
H	-1.74883900	1.51387600	-2.22299300

H	-0.18874900	-2.40684100	0.55763400
H	-2.89032100	1.73709500	0.61621100
H	-3.76679600	1.98847100	-0.90854700
H	1.55110000	2.34195500	-1.33672700
H	2.59999400	-2.22281500	0.48875200
H	-2.75150000	-2.75156900	0.92581400
H	-2.58441000	-3.01062000	-0.81335600
H	-4.13079900	-2.43122400	-0.14493300
H	3.89826800	1.60232800	-0.99306400
H	4.79666100	-1.51480400	0.24403800
H	-4.38184600	-0.20185400	-0.46100700
O	0.24795200	0.73025900	2.46941100
O	-0.79831400	1.45960000	2.18996900
H	0.96982900	1.03872100	1.88596300

monocation RAF TS

O	-4.71052200	0.63516600	-0.17532500
N	0.09174300	1.49591800	0.09957100
N	3.36336600	0.01812400	-0.42687500
C	0.66517400	-0.66178100	-0.33457400
C	1.14504600	0.65371300	-0.10984400
C	1.60017400	-1.65813500	-0.94255500
C	-0.76498400	-0.53925100	-0.42004000
C	-1.08574700	0.79257800	-0.08278800
C	3.01609700	-1.41397000	-0.42047200
C	2.51247000	0.99522900	-0.22432800
C	-1.79688200	-1.45795100	-0.65250600
C	-2.39838600	1.24623400	0.02039100
C	2.98263500	2.40667200	-0.16154200
C	-3.10255300	-1.02576800	-0.56494300
C	-3.39680600	0.31550100	-0.23236200
H	1.30387700	-2.67791000	-0.69486800
H	1.56329700	-1.55145500	-2.03089300
H	0.14904100	2.46875400	0.36787800
H	3.11010500	-1.76500500	0.60838900
H	3.75187100	-1.92629600	-1.03594600
H	-1.57379100	-2.48976400	-0.89704500

H	-2.63071800	2.27244300	0.27922100
H	2.74264100	2.82505700	0.81879900
H	2.46753000	2.99964900	-0.91984400
H	4.05679200	2.47474800	-0.32161000
H	-3.93021400	-1.69981100	-0.74517700
H	-4.82695100	1.56342000	0.05622100
H	4.34427700	0.25692100	-0.51924100
O	0.07020100	-0.60203100	2.36731600
O	0.87131800	-1.31992400	1.53749400
H	-0.81891900	-0.97316100	2.24274900

monocation RAF PC

O	-4.74363400	0.70284400	-0.26083100
N	0.07253100	1.49802500	0.13012100
N	3.31731600	0.05700300	-0.48208600
C	0.66148800	-0.76265800	-0.03225400
C	1.13331000	0.67292200	0.04332600
C	1.46673500	-1.47153600	-1.11455200
C	-0.82541900	-0.57806300	-0.18930100
C	-1.12494500	0.77478700	-0.02740200
C	2.95494300	-1.33217100	-0.79407300
C	2.47151500	1.02090000	-0.12819300
C	-1.85312600	-1.48980900	-0.35732700
C	-2.41663300	1.26994700	-0.04192200
C	2.96227900	2.42469300	0.01399400
C	-3.16559700	-1.02760700	-0.38187400
C	-3.43977500	0.33517000	-0.22573300
H	1.20207000	-2.52896800	-1.15790500
H	1.23102100	-1.01554100	-2.07799900
H	0.12045300	2.50934500	0.14689600
H	3.22233000	-1.95449900	0.06183400
H	3.55924000	-1.63932600	-1.64654800
H	-1.64606800	-2.54783400	-0.46966300
H	-2.62910500	2.32527500	0.08187900
H	2.61312400	3.02357600	-0.83124000
H	4.05054100	2.45667300	0.03225800
H	2.58240800	2.86986300	0.93433000

H	-3.99483800	-1.70942300	-0.51990200
H	-4.83428700	1.65391000	-0.13600300
H	4.29668100	0.30164700	-0.56300500
O	0.48710200	-0.77750200	2.28831300
O	0.99490000	-1.47889400	1.16827800
H	-0.35810200	-1.22093900	2.45460900

neutral RAF RC

O	-4.61374400	0.83075800	0.04683000
N	0.21062300	1.58278700	0.40556600
N	3.54462100	0.14585700	-0.20476500
C	0.76849900	-0.32195000	-0.66478100
C	1.25684700	0.78353100	-0.02489200
C	1.73530400	-1.28531800	-1.27464800
C	-0.66051500	-0.22518500	-0.62838800
C	-0.96914700	0.97721600	0.06471700
C	3.03382100	-1.20906000	-0.46481000
C	2.69217600	1.06488900	0.04915400
C	-1.71736700	-1.03879300	-1.08233100
C	-2.28844500	1.37496600	0.32132800
C	3.13831300	2.45387500	0.40156100
C	-3.01588100	-0.65180300	-0.83827700
C	-3.29437300	0.54762600	-0.14205300
H	1.35534700	-2.30822100	-1.24791000
H	1.91501900	-1.02594700	-2.32523100
H	0.29430100	2.42840200	0.94930900
H	2.86044600	-1.67711300	0.51409400
H	3.82515200	-1.77343000	-0.95852300
H	-1.50952100	-1.96209400	-1.61070300
H	-2.51211100	2.29029800	0.85734100
H	2.72088700	3.18133400	-0.29934300
H	4.22490200	2.51023100	0.37959200
H	2.78960000	2.72324300	1.40293000
H	-3.85254900	-1.25518500	-1.16807700
H	-4.71109800	1.65982500	0.52709500
O	-0.30830600	-1.87193700	1.96452500
O	0.28012200	-2.77470500	1.23065500

H	-0.62704200	-1.18474500	1.33827100

neutral RAF TS

O	-4.68392500	0.66947600	-0.16650800
N	0.13667600	1.48531900	0.13653400
N	3.47905500	0.08627100	-0.43140100
C	0.70590100	-0.65841800	-0.33594000
C	1.18692600	0.64221500	-0.08920800
C	1.65481500	-1.61062700	-0.98974000
C	-0.73452500	-0.52884100	-0.42735700
C	-1.04980300	0.79570600	-0.06348300
C	3.07391400	-1.32193700	-0.47858600
C	2.59240900	0.98636600	-0.20581900
C	-1.77011800	-1.43173500	-0.67907700
C	-2.36074300	1.25196900	0.04637900
C	2.98713300	2.43142700	-0.09285300
C	-3.08054000	-0.99614100	-0.58794400
C	-3.36709500	0.33389900	-0.22889500
H	1.39019900	-2.64749300	-0.77291800
H	1.60028100	-1.47356600	-2.07549700
H	0.20266400	2.45841400	0.39757400
H	3.16905500	-1.70910000	0.54115400
H	3.80299200	-1.85237800	-1.09208100
H	-1.55272000	-2.46067900	-0.94243400
H	-2.58975200	2.27362100	0.32724100
H	2.46094200	3.03520300	-0.83696200
H	4.06011300	2.53129700	-0.24387500
H	2.72933400	2.82197900	0.89560800
H	-3.90907100	-1.66443600	-0.78610800
H	-4.78395500	1.59341600	0.08663400
O	0.05371200	-0.74855100	2.34400700
O	0.87011400	-1.44602700	1.47427300
H	-0.83903400	-1.06988200	2.14670000

neutral RAF PC

O	-4.69776500	0.72818400	-0.27853900
N	0.11199000	1.48072000	0.26004200
N	3.42286300	0.15696000	-0.57534300

C	0.70383200	-0.77012700	-0.03265400
C	1.19016600	0.64599500	0.10538100
C	1.52887800	-1.44991300	-1.11537600
C	-0.78027500	-0.56821800	-0.20646300
C	-1.07395700	0.77957200	0.03016000
C	3.01569500	-1.22755100	-0.80366800
C	2.53024300	1.01414600	-0.15786000
C	-1.80815900	-1.46352700	-0.44814200
C	-2.37239600	1.26813800	0.01876600
C	2.92773600	2.45748800	0.01180800
C	-3.12236300	-1.00269700	-0.47275500
C	-3.39208100	0.34943200	-0.24067100
H	1.30146400	-2.51750000	-1.16330900
H	1.26736700	-0.99987100	-2.07638400
H	0.16828200	2.48875900	0.27088800
H	3.28636700	-1.81023100	0.08447900
H	3.62038800	-1.61557900	-1.62599700
H	-1.59759700	-2.51366600	-0.61934300
H	-2.59164400	2.31427000	0.19993600
H	2.39094900	3.09166900	-0.70021200
H	3.99605000	2.56395200	-0.16744300
H	2.69638200	2.81563000	1.01814900
H	-3.94978100	-1.67276200	-0.66796700
H	-4.78060300	1.67010200	-0.09545700
O	0.44370300	-0.93377400	2.29305200
O	0.98221500	-1.56890500	1.14699500
H	-0.46073200	-1.27900600	2.32655700

zwitterion I RC

O	-4.68333700	0.68731200	0.07032600
N	0.09629500	1.58006000	0.44808500
N	3.36601600	0.22247600	-0.36588300
C	0.68501000	-0.36018900	-0.70615100
C	1.11774200	0.79475600	-0.02988400
C	1.66477400	-1.27054800	-1.37745200
C	-0.71664500	-0.29672900	-0.65218900
C	-1.01226500	0.91798200	0.08272000

C	2.99207400	-1.17698700	-0.63010000
C	2.49379100	1.13260100	0.00798000
C	-1.77661300	-1.11800500	-1.12485900
C	-2.36721800	1.26736900	0.34720400
C	2.96217300	2.48750700	0.41061100
C	-3.06627700	-0.75104100	-0.86131500
C	-3.35259100	0.44217900	-0.12505900
H	1.33380200	-2.31061000	-1.36151600
H	1.79252200	-0.98199000	-2.42756400
H	2.89666200	-1.67468000	0.34036700
H	3.80661400	-1.63588100	-1.18621300
H	-1.56555700	-2.02530900	-1.68039600
H	-2.60616400	2.16787200	0.90376800
H	4.03782400	2.51107300	0.57820800
H	2.43370000	2.79048700	1.31458200
H	2.70119900	3.20206500	-0.37320800
H	-3.90407900	-1.35017900	-1.19722500
H	-4.79490300	1.50398800	0.56836400
H	4.34989800	0.45871600	-0.35281300
O	-0.11025600	-1.56603400	2.08231500
O	0.78206700	-2.44570700	1.72134400
H	-0.36922900	-1.09124500	1.25732500

zwitterion I RAF TS

O	-4.68868900	0.67576200	-0.13231000
N	0.10432400	1.56390200	0.14661300
N	3.35952700	0.08064900	-0.39370700
C	0.67660700	-0.62938400	-0.41089900
C	1.11083500	0.69529100	-0.10095600
C	1.63951500	-1.60428300	-1.00639900
C	-0.73999800	-0.51478000	-0.47392600
C	-1.02784300	0.82556100	-0.06794000
C	3.02773900	-1.35390800	-0.41748200
C	2.48294800	1.03753100	-0.17356100
C	-1.78537200	-1.41638800	-0.73348400
C	-2.36241800	1.25813500	0.06063700
C	2.94260500	2.45102000	-0.07355500

C	-3.08737500	-0.98115600	-0.61138600
C	-3.36554400	0.35172900	-0.21826900
H	1.35120800	-2.63516100	-0.79297400
H	1.65694500	-1.48234600	-2.09522300
H	3.06128700	-1.71421600	0.61243400
H	3.80110200	-1.85608000	-0.99529700
H	-1.57667300	-2.44041800	-1.02387500
H	-2.59120600	2.27316000	0.36825400
H	4.02424000	2.51666200	0.03510800
H	2.45184000	2.92814700	0.77474300
H	2.63654200	2.99054300	-0.97264300
H	-3.92320500	-1.64125500	-0.80855900
H	-4.78582400	1.59416500	0.14096700
H	4.33762300	0.33365400	-0.45764800
O	0.01192200	-0.72421100	2.36501400
O	0.81803600	-1.46793600	1.54276600
H	-0.89117200	-0.96384200	2.10394900

zwitterion I RAF PC

O	-4.72998400	0.73582000	-0.24049400
N	0.07062500	1.58108600	0.12964800
N	3.32279400	0.09463800	-0.44127000
C	0.67063300	-0.74169800	-0.05240700
C	1.09267700	0.71670500	0.05678500
C	1.49657100	-1.42028700	-1.13961800
C	-0.81449700	-0.56589500	-0.21129500
C	-1.07940800	0.80786000	-0.02235600
C	2.97963200	-1.28444500	-0.78378200
C	2.43547300	1.05154400	-0.08774300
C	-1.84512700	-1.47295900	-0.37838100
C	-2.39176100	1.28161300	-0.02986800
C	2.94255700	2.45062800	0.06373200
C	-3.16243900	-1.00839500	-0.39179000
C	-3.42293000	0.35719600	-0.21785500
H	1.23855800	-2.47933200	-1.22696400
H	1.27982200	-0.93301900	-2.09435000
H	3.21465300	-1.94034900	0.06063100

H	3.59991700	-1.58697800	-1.63043400
H	-1.64198300	-2.53340000	-0.50296700
H	-2.60340600	2.33794100	0.10896700
H	3.73125600	2.49452700	0.82136400
H	2.12550400	3.10585200	0.36219000
H	3.36477200	2.81195600	-0.87908100
H	-3.99892200	-1.68370600	-0.53126500
H	-4.79415100	1.68983500	-0.10626300
H	4.29760400	0.35381200	-0.49560100
O	0.42603000	-0.88063400	2.26776600
O	0.99403200	-1.50697100	1.12733000
H	-0.49648200	-1.18413600	2.22578100

monoanion RAF RC

O	-4.68302700	0.69441700	0.12354600
N	0.12856800	1.54422300	0.43398000
N	3.46237900	0.22489500	-0.35970000
C	0.69392500	-0.37581900	-0.73900100
C	1.13525000	0.76811500	-0.06898900
C	1.69131500	-1.25272000	-1.43093100
C	-0.72402300	-0.30839200	-0.67933000
C	-1.00428300	0.88766400	0.07149300
C	3.01272000	-1.14140900	-0.66210400
C	2.56683900	1.08686800	-0.03717900
C	-1.78593400	-1.11850500	-1.12942400
C	-2.34532800	1.24038000	0.36067900
C	2.97698000	2.47723700	0.35674800
C	-3.08411400	-0.75133600	-0.84740900
C	-3.35242400	0.42542000	-0.10414900
H	1.38077300	-2.30085500	-1.45851600
H	1.83136300	-0.93199400	-2.47234000
H	2.89877900	-1.66429800	0.29901600
H	3.81581600	-1.64468400	-1.20322100
H	-1.58899500	-2.02555800	-1.69223900
H	-2.57185800	2.13615300	0.93195000
H	2.55046700	3.20950800	-0.33325300
H	4.06261200	2.56279000	0.35716700

H	2.58743200	2.71284100	1.34992200
H	-3.92394200	-1.35009400	-1.18016200
H	-4.76261300	1.51292900	0.62355900
O	0.43715100	-1.29627000	2.10313200
O	0.40950400	-2.57164400	1.83387700
H	0.58897700	-0.83759800	1.23129900

monoanion I RAF TS

O	-4.67201600	0.71653400	-0.12806700
N	0.14344800	1.56483500	0.16587300
N	3.46769300	0.15614000	-0.39783300
C	0.70846800	-0.63152600	-0.36273400
C	1.14159900	0.69746600	-0.07233000
C	1.67299100	-1.53421800	-1.06091600
C	-0.72516300	-0.50965100	-0.44412500
C	-1.00697700	0.83037600	-0.04676400
C	3.08261700	-1.25380800	-0.52262300
C	2.56032400	1.03299500	-0.15056300
C	-1.76917400	-1.39992800	-0.70503500
C	-2.33353700	1.26879700	0.07364300
C	2.95623400	2.47293700	0.02607900
C	-3.08047100	-0.95911500	-0.59706500
C	-3.34911800	0.36714900	-0.20981300
H	1.42572100	-2.58797900	-0.90948300
H	1.63366700	-1.33862600	-2.14000300
H	3.16897200	-1.69844400	0.47447500
H	3.82671000	-1.74104000	-1.15627100
H	-1.56473100	-2.42753000	-0.98849000
H	-2.56002000	2.28640600	0.37702800
H	2.47756100	3.09567200	-0.73339200
H	4.03811200	2.57311000	-0.04827600
H	2.61842400	2.84071700	0.99792700
H	-3.91538800	-1.61823000	-0.80180400
H	-4.74901400	1.63603800	0.14643900
O	0.04216700	-0.92693300	2.32901600
O	0.88224200	-1.57682500	1.40888300
H	-0.84389600	-1.05798000	1.96038800

monoanion I RAF PC

O	-4.68937100	0.75328900	-0.23866700
N	0.11268200	1.56276900	0.20038500
N	3.43066700	0.18691100	-0.51618700
C	0.70798000	-0.74663600	-0.05922800
C	1.14759200	0.69086600	0.09485500
C	1.55441300	-1.41265300	-1.13307600
C	-0.77261700	-0.55829200	-0.23029600
C	-1.02824300	0.81665500	0.01382900
C	3.03318900	-1.19378200	-0.78120300
C	2.50831800	1.03319100	-0.11375400
C	-1.80285200	-1.45045800	-0.45462700
C	-2.35369300	1.27917700	0.01803500
C	2.91610000	2.47404100	0.07224300
C	-3.12385000	-0.99144700	-0.46272400
C	-3.37585800	0.36498500	-0.22357900
H	1.33090900	-2.48054400	-1.21350900
H	1.31575200	-0.94191900	-2.09088400
H	3.27635400	-1.80491700	0.09743600
H	3.65673200	-1.56956500	-1.59755100
H	-1.59695000	-2.50326200	-0.62511500
H	-2.57806800	2.32553400	0.20145900
H	2.45934500	3.10453400	-0.69598000
H	4.00008600	2.56124400	0.00083000
H	2.58221700	2.85685200	1.03930800
H	-3.95624600	-1.65910500	-0.64511000
H	-4.75083500	1.69718200	-0.05959400
O	0.39788700	-0.99442600	2.27258400
O	0.98437800	-1.57260000	1.11839200
H	-0.54876400	-1.15777800	2.13799800

monoanion II RAF RC

O	4.59311400	-0.92992400	0.03738300
N	-0.22151000	-1.54228400	0.40970100
N	-3.54349800	-0.05904000	-0.24039000
C	-0.75060400	0.35819000	-0.68337900
C	-1.26372800	-0.71902700	-0.01188400

C	-1.70060100	1.32655100	-1.31142600
C	0.67367200	0.22781200	-0.67082600
C	0.97574700	-0.96842200	0.04536200
C	-3.00523500	1.28274200	-0.50834500
C	-2.69452100	-0.97834300	0.04739700
C	1.75422200	1.00387000	-1.15591400
C	2.28082100	-1.38419300	0.31192900
C	-3.16344700	-2.35479800	0.42482600
C	3.04097200	0.60004700	-0.90627200
C	3.37421000	-0.60937700	-0.16379000
H	-1.30727300	2.34555200	-1.30065900
H	-1.87654600	1.05670800	-2.36095100
H	-0.31317200	-2.34706900	1.00959300
H	-2.81800400	1.75523200	0.46658200
H	-3.78183000	1.86266500	-1.00845300
H	1.55942400	1.91945800	-1.70621000
H	2.47874900	-2.29219800	0.87111800
H	-2.74312000	-3.10384700	-0.25121400
H	-4.25037900	-2.40053600	0.38773600
H	-2.83264800	-2.60474900	1.43736900
H	3.87704700	1.19192600	-1.26458100
O	0.65759500	1.63444500	1.96696400
O	-0.20147800	2.54722100	1.60498000
H	0.83989500	1.10188100	1.15382100

monoanion II RAF TS

O	-4.68437000	0.77389900	-0.13255100
N	0.11829900	1.49696600	0.12970200
N	3.44790500	0.05103500	-0.41931400
C	0.65498500	-0.64965800	-0.36463100
C	1.16101200	0.64314300	-0.09165400
C	1.60343400	-1.61542100	-0.99670600
C	-0.76741000	-0.50786200	-0.45551000
C	-1.08542600	0.82071900	-0.07928700
C	3.01772400	-1.34992100	-0.46056300
C	2.56598400	0.96313600	-0.19926000
C	-1.82885200	-1.40092400	-0.69369600

C	-2.37872200	1.29296900	0.04270000
C	2.98363800	2.40261600	-0.08150600
C	-3.12684000	-0.95932500	-0.58480400
C	-3.47219900	0.40337800	-0.21778400
H	1.31891100	-2.64889400	-0.78894500
H	1.57365600	-1.47604700	-2.08396700
H	0.19441500	2.46375900	0.40794500
H	3.08040800	-1.72979900	0.56475200
H	3.74900300	-1.90130600	-1.05350100
H	-1.62052100	-2.43303400	-0.96031900
H	-2.58754800	2.31776600	0.32960800
H	2.46907900	3.01774500	-0.82448900
H	4.05873100	2.48727300	-0.22824000
H	2.72868500	2.79479900	0.90722100
H	-3.95319900	-1.63655700	-0.77323300
O	-0.06359600	-0.76997700	2.32280300
O	0.87031400	-1.41958300	1.52052300
H	-0.91779700	-1.02350700	1.94045600

monoanion II RAF PC

O	-4.70215400	0.84824500	-0.27733900
N	0.09256800	1.48417700	0.27512900
N	3.39900900	0.14048300	-0.56158800
C	0.66168000	-0.76902700	-0.04092100
C	1.16024600	0.64362900	0.10475400
C	1.49994100	-1.45598700	-1.10997400
C	-0.81333000	-0.55598800	-0.21985700
C	-1.11155400	0.79154600	0.02855000
C	2.98532700	-1.24336500	-0.78500000
C	2.50210700	1.00125500	-0.15022900
C	-1.86846900	-1.43291600	-0.46174800
C	-2.39324900	1.30222400	0.02114400
C	2.90558400	2.44435700	0.01551600
C	-3.17150300	-0.95931300	-0.48241500
C	-3.49963300	0.42479900	-0.24875800
H	1.26631800	-2.52243800	-1.16069100
H	1.25252200	-1.00595600	-2.07484500

H	0.16013200	2.49121700	0.24785900
H	3.24114500	-1.82365800	0.10953100
H	3.59691200	-1.64176100	-1.59789800
H	-1.67100900	-2.48694400	-0.63883400
H	-2.58773400	2.35257500	0.21053800
H	2.38612800	3.07713800	-0.71079400
H	3.97781600	2.54422000	-0.14451600
H	2.65759200	2.81274900	1.01434400
H	-3.99507700	-1.63704700	-0.68098400
O	0.35319200	-0.96993000	2.28702500
O	0.93980200	-1.57331100	1.14774600
H	-0.59539500	-1.12002800	2.14566800

monocation PCET RC

O	-3.78133100	-1.27202600	-0.03345500
N	0.48807900	1.11303500	0.04582400
N	4.11482000	0.63199100	0.05052400
C	1.72739800	-0.79164000	-0.01298400
C	1.78258900	0.60143000	0.04881600
C	2.99220800	-1.57982500	-0.15948300
C	0.36191400	-1.14987400	-0.04613200
C	-0.39119900	0.06657100	0.01306600
C	4.15326400	-0.78869200	0.44934600
C	2.99343500	1.32217600	-0.05280700
C	-0.33172400	-2.38091200	-0.11211500
C	-1.79035900	0.07960100	0.02825800
C	3.04756700	2.79545200	-0.28575100
C	-1.70476000	-2.37349400	-0.10574600
C	-2.43251700	-1.15057900	-0.03447300
H	2.93034300	-2.54715700	0.34242500
H	3.17257100	-1.78354000	-1.22242200
H	0.23361700	2.08214200	0.15840300
H	4.09730700	-0.82079800	1.54310600
H	5.11617200	-1.19199400	0.14228300
H	0.21096200	-3.31732000	-0.16173800
H	-2.34800700	1.00705100	0.07884500
H	2.46824900	3.06165600	-1.17264300
H	4.07298100	3.14156800	-0.41242400
H	2.60799000	3.31872000	0.56845800
H	-2.26946800	-3.29650600	-0.15182100
H	-4.22258200	-0.40239900	0.01134700
H	4.99422300	1.12695500	-0.02380600
O	-4.66493500	2.23415500	0.08578500
O	-5.35468300	1.10615800	0.08651800
H	-5.32396500	2.95884300	0.13479400

monocation PCET TS

O	-3.82450800	-0.99367900	-0.14725400
N	0.53315300	1.15529500	-0.05701300
N	4.10673200	0.48938500	0.12750700
C	1.65617600	-0.82040000	-0.01376700
C	1.78245800	0.57866500	-0.00759600

C	2.88404400	-1.67330500	-0.07342200
C	0.28454500	-1.10423200	-0.06385800
C	-0.40710700	0.15328900	-0.07208700
C	4.05599100	-0.92204700	0.56397500
C	3.04239900	1.23618500	-0.06796800
C	-0.47320800	-2.30929200	-0.09729000
C	-1.78935600	0.24693400	-0.09615700
C	3.18714100	2.69137600	-0.35608300
C	-1.83557800	-2.23648800	-0.12214100
C	-2.52060500	-0.96899500	-0.11998600
H	2.74802200	-2.61884400	0.45355800
H	3.10451900	-1.91780000	-1.11930100
H	0.32911600	2.14294500	-0.01215800
H	3.95981700	-0.91663800	1.65444100
H	5.00936100	-1.37741900	0.30543400
H	0.02858200	-3.26926300	-0.09760900
H	-2.30631400	1.19774800	-0.09656400
H	2.69290900	2.94179600	-1.29756800
H	4.23466300	2.98395700	-0.41270500
H	2.70685900	3.27183900	0.43703200
H	-2.44782900	-3.12918000	-0.14186300
H	-4.38836100	-0.03070100	-0.02883900
H	5.01728400	0.93153600	0.08114200
O	-4.39679400	2.08516700	0.26792900
O	-5.19913700	0.94482800	0.16987400
H	-5.00570000	2.75659900	0.61544400

monocation PCET PC

O	-3.74534500	-1.11178500	0.07558800
N	0.52764100	1.13755300	0.08659900
N	4.11172800	0.57172500	0.04303800
C	1.69405100	-0.80923300	-0.05796400
C	1.78279900	0.59860700	0.02556900
C	2.94154300	-1.61398700	-0.24426600
C	0.33880300	-1.12967200	-0.03887000
C	-0.39081700	0.10782100	0.06215300
C	4.13042700	-0.87055700	0.37130900
C	3.02300900	1.29939600	-0.04677500
C	-0.38263700	-2.36767900	-0.09406600
C	-1.76324500	0.16016300	0.10786900
C	3.11193700	2.77520100	-0.22891000
C	-1.73778800	-2.34917500	-0.04624000
C	-2.49083500	-1.09649600	0.04953500
H	2.86564700	-2.59721300	0.22156100
H	3.10592200	-1.77788100	-1.31545000
H	0.29963600	2.11628300	0.18889000
H	4.11251100	-0.94784400	1.46257000
H	5.07664500	-1.27092500	0.01472200
H	0.15873500	-3.30283900	-0.16952800
H	-2.32507500	1.08453200	0.17689800
H	2.55430000	3.08320900	-1.11779800
H	4.14736800	3.10084800	-0.32381800
H	2.66673200	3.27803100	0.63617900
H	-2.31999500	-3.26153700	-0.08177500
H	-4.79868500	0.26444100	-0.08307300
H	5.00658300	1.04490500	-0.01722500
O	-4.54686200	2.15333600	0.07689100
O	-5.41481900	1.02491700	-0.22412100
H	-4.91318100	2.46295800	0.91745500

neutral PCET RC

O	-4.24302400	-1.18144600	0.00032600
N	0.12915600	1.05615700	0.05772100
N	3.79456800	0.56942300	0.10781800
C	1.31521900	-0.86618700	-0.02348400
C	1.40759100	0.50559400	0.04385000
C	2.57589600	-1.66328000	-0.16060600
C	-0.07594700	-1.19650800	-0.04834800
C	-0.79075300	0.03621900	0.02167600
C	3.72702300	-0.85793000	0.46756900
C	2.68329900	1.20592700	-0.03628500
C	-0.80978600	-2.39628600	-0.10965000
C	-2.18976800	0.09271700	0.04718000
C	2.69833500	2.68596800	-0.31213500
C	-2.19170000	-2.35002100	-0.09264600
C	-2.87710500	-1.11437400	-0.01322100
H	2.50720900	-2.63555100	0.33506200
H	2.77892900	-1.86709800	-1.22170900
H	-0.09278000	2.03399500	0.15967000
H	3.63278900	-0.90487600	1.56252900
H	4.68764300	-1.31163100	0.21545400
H	-0.30056100	-3.35192600	-0.16573800
H	-2.71668500	1.03832300	0.10601600
H	2.17692600	2.91950000	-1.24547900
H	3.72624000	3.04060700	-0.37841500
H	2.19122600	3.23398100	0.48934000
H	-2.77917700	-3.25942700	-0.13728000
H	-4.63672500	-0.29086700	0.04168200
O	-4.98771100	2.39100700	0.09644500
O	-5.72524700	1.29414500	0.09402100
H	-5.61507600	3.14384700	0.09863900

neutral PCET TS

O	-3.79992600	-1.07934500	-0.02144400
N	0.55723800	1.12795500	0.02225900
N	4.19455300	0.60040400	0.13108800
C	1.70961700	-0.81695800	-0.05329800
C	1.81461000	0.57240000	0.00229000
C	2.96395400	-1.62167500	-0.16427200
C	0.33271900	-1.12997200	-0.06746200
C	-0.37832000	0.11192700	-0.00642900
C	4.11848700	-0.82760800	0.47385500
C	3.10053500	1.25769300	-0.04298700
C	-0.41040600	-2.33538000	-0.11947800
C	-1.76213900	0.18550400	0.01547100
C	3.14468900	2.73808000	-0.30687700
C	-1.78218700	-2.28064200	-0.10332000
C	-2.47184000	-1.03224500	-0.03473400
H	2.87450500	-2.59403000	0.32609900

H	3.18031500	-1.82186000	-1.22253100
H	0.34391300	2.11161900	0.09086000
H	4.03218600	-0.88256800	1.56811500
H	5.07571700	-1.28240600	0.21348700
H	0.09869200	-3.29077400	-0.16909000
H	-2.29015600	1.12986500	0.06461600
H	2.65044500	2.98564200	-1.25113200
H	4.18005000	3.07380100	-0.34647400
H	2.62964800	3.28898100	0.48715400
H	-2.37954200	-3.18331200	-0.14054800
H	-4.27181600	-0.16141500	0.02690600
O	-4.36588700	2.15638600	0.12593800
O	-5.16647200	1.01255100	0.09039000
H	-5.00653000	2.88447200	0.16349700

neutral PCET PC

O	-4.16733400	-1.01499000	0.20710000
N	0.19125600	1.10566200	0.12399100
N	3.81009800	0.50709900	0.05151700
C	1.29415200	-0.86513100	-0.04600700
C	1.42860000	0.53249300	0.02640300
C	2.52701800	-1.68422300	-0.24589200
C	-0.07231700	-1.15294200	0.01623200
C	-0.76827800	0.10352900	0.12714600
C	3.72999600	-0.92970100	0.34844600
C	2.72472300	1.19313200	-0.05637400
C	-0.83146700	-2.36269800	-0.00631400
C	-2.13589400	0.19496500	0.19892000
C	2.79134200	2.68104400	-0.26888900
C	-2.18903600	-2.30098300	0.07392200
C	-2.90290000	-1.03030600	0.16550900
H	2.44550600	-2.67140900	0.21437000
H	2.68124300	-1.84810900	-1.32040400
H	-0.00172600	2.09219600	0.21494100
H	3.71280600	-1.02083200	1.44271100
H	4.66149100	-1.38801400	0.01360000
H	-0.32317200	-3.31699000	-0.08536600
H	-2.66435000	1.13895200	0.26678000
H	2.24121100	2.97968500	-1.16846400
H	3.83258600	2.98901800	-0.36628900
H	2.34788300	3.21419000	0.58164400
H	-2.79606200	-3.19834100	0.05974500
H	-5.16056500	0.28377500	-0.22256200
O	-5.03669800	2.19509600	-0.24256700
O	-5.78267600	0.98500700	-0.55392200
H	-5.58743400	2.58882400	0.45007100

zwitterion 1 PCET RC

O	-3.74287500	-1.16636300	-0.23017800
N	0.53338300	1.20303200	-0.11117000

N	4.14724200	0.65978200	0.11935700
C	1.77374800	-0.79080400	0.02844900
C	1.79270900	0.63253300	-0.02336200
C	3.05579700	-1.57008100	0.00167200
C	0.42092100	-1.13272500	-0.04234900
C	-0.29317000	0.14065500	-0.11090300
C	4.16676600	-0.71953400	0.63089400
C	3.00798000	1.33452000	-0.07564400
C	-0.30880800	-2.35303200	-0.05257200
C	-1.71505900	0.14652100	-0.16818300
C	3.08382600	2.79713600	-0.38632100
C	-1.67961300	-2.31284300	-0.11600500
C	-2.38266500	-1.06207100	-0.17328500
H	2.98667200	-2.51307000	0.55367100
H	3.32562900	-1.83240300	-1.03283500
H	4.02564800	-0.68084600	1.72020400
H	5.15442400	-1.13706000	0.42971700
H	0.20776700	-3.30870400	-0.00598300
H	-2.25949700	1.08616300	-0.21176000
H	3.41802600	2.94439500	-1.42008100
H	3.79720200	3.30522200	0.27014800
H	2.09549900	3.24320700	-0.27831700
H	-2.27091700	-3.22335700	-0.12204800
H	-4.14350000	-0.27592300	-0.27404100
H	5.01812200	1.17370600	0.12186900
O	-5.41464900	1.65692800	0.88344800
O	-5.06215500	1.38510500	-0.36831500
H	-5.89660700	2.51197300	0.83816800

zwitterion 1 PCET TS

O	-3.77477300	-1.13742300	0.13055900
N	0.50912300	1.18420700	0.15664300
N	4.10366100	0.64703100	0.01856900
C	1.72857800	-0.79905400	-0.05175900
C	1.75430100	0.62108100	0.06441900
C	2.99501200	-1.56387500	-0.27718900
C	0.37282800	-1.13709900	-0.03905700

C	-0.33544300	0.12236500	0.10601100
C	4.16497900	-0.79109900	0.34080600
C	2.97220400	1.33617400	-0.02093200
C	-0.35885600	-2.34969800	-0.11410200
C	-1.74087600	0.14056400	0.18786700
C	3.02393400	2.81733600	-0.19999300
C	-1.73136600	-2.31157600	-0.04907700
C	-2.42668100	-1.07223400	0.08878800
H	2.96415200	-2.56233300	0.16492100
H	3.16060200	-1.70084700	-1.35440900
H	4.13895300	-0.88486300	1.43239200
H	5.12396800	-1.16737600	-0.01173600
H	0.15322600	-3.29989300	-0.22204700
H	-2.28273800	1.06786200	0.32840500
H	2.76759800	3.06931500	-1.23320400
H	4.01231200	3.21917300	0.02438900
H	2.27935300	3.29040000	0.44062200
H	-2.32091700	-3.21861600	-0.10986000
H	-4.16219900	-0.23593400	-0.05246400
H	4.97869200	1.15064400	-0.04084900
O	-5.29031000	1.88804400	0.40003400
O	-4.67790200	1.21170700	-0.62878700
H	-5.67029800	2.67787600	-0.02178200

zwitterion 1 PCET PC

O	-3.69010400	-0.96212900	-0.21912500
N	0.61485400	1.22988900	-0.11993500
N	4.16404700	0.59668500	0.16740100
C	1.75877900	-0.80421300	0.00019200
C	1.82602600	0.63574200	-0.04328200
C	3.01760400	-1.61033300	-0.01856700
C	0.41219000	-1.10484600	-0.05340400
C	-0.27287500	0.17774100	-0.11665500
C	4.14704600	-0.80408100	0.63274400
C	3.07576500	1.30994800	-0.06882700
C	-0.35082300	-2.31502100	-0.05897800
C	-1.65364100	0.24465600	-0.16994200

C	3.19894700	2.76321600	-0.38160700
C	-1.70963300	-2.25075500	-0.11443000
C	-2.42424900	-0.97775900	-0.17083700
H	2.91238500	-2.56059300	0.50868200
H	3.28242700	-1.84978200	-1.05647600
H	4.02012900	-0.78671600	1.72033800
H	5.12178700	-1.23435100	0.41021400
H	0.15185900	-3.27565000	-0.01588400
H	-2.17572800	1.19347800	-0.21860800
H	2.92970100	2.93865400	-1.42648600
H	4.21071100	3.12968800	-0.20813600
H	2.49488000	3.32932300	0.23057600
H	-2.31684700	-3.14845400	-0.11605700
H	-4.66213300	0.38944100	-0.33329100
H	5.05991900	1.06741200	0.15105500
O	-5.47301000	1.56654900	0.95206400
O	-5.29721700	1.15251800	-0.43034000
H	-4.94957100	2.38024500	0.98256900
